# RNA-binding protein transcripts reflect composition of target mRNAs

**DOI:** 10.1093/nar/gkag540

**Published:** 2026-06-08

**Authors:** Thomas H Kapral, Bojan Žagrović

**Affiliations:** Max Perutz Labs, Vienna BioCenter, 1030 Vienna, Austria; University of Vienna, 1030 Vienna, Austria; Vienna BioCenter PhD Program, a Doctoral School of the University of Vienna and the Medical University of Vienna, 1030 Vienna, Austria; Max Perutz Labs, Vienna BioCenter, 1030 Vienna, Austria; University of Vienna, 1030 Vienna, Austria

## Abstract

RNA–protein interactions are central to gene regulation, yet the large-scale organization of RNA-protein networks remains incompletely understood. Using a comprehensive human eCLIP dataset encompassing interactions between 150 RNA-binding proteins (RBPs) and >11 000 mRNAs, we identify a robust organizing principle underlying the RNA–protein network structure: mRNAs preferentially associate with RBPs whose own encoding transcripts share similar nucleotide composition. In other words, mRNAs enriched in a given nucleotide tend to be targeted by RBPs whose own transcripts are likewise enriched in that nucleotide and vice versa. This global pattern holds for all four RNA nucleotides, remains statistically significant after controlling for transcript length, expression levels and sequence-motif-driven interactions, and is confirmed by *in vitro* HTR-SELEX data. We use the observed relationship to rationalize the spatial organization of mRNAs in the nucleus i.e. the known G/C gradient towards nuclear speckles. Notably, an mRNA’s propensity toward RBPs rich in arginine, which is predominantly encoded by and preferentially binds guanine, is a strong predictor of its speckle enrichment. Our findings highlight a fundamental link between coding and binding in biology and suggest that mRNA composition biases provide a fundamental layer of specificity in shaping the global RNA–protein interaction network.

## Graphical Abstract

**Graphical Abstract:**
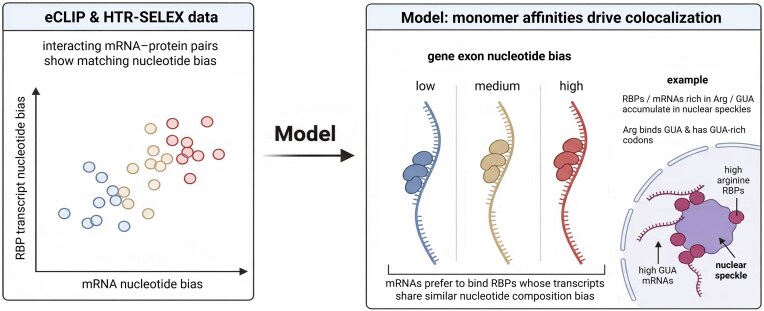

## Introduction

Direct, noncovalent interactions between RNA and proteins play an important role in various cellular processes involved in gene expression [1–5]. Moreover, dysfunction in RNA–protein interactions has been implicated in different diseases, underscoring their significance in maintaining cellular homeostasis [6–11]. A hallmark of intracellular RNA-protein interaction networks is their extreme complexity. In particular, proteome-wide approaches have identified over 3000 RNA-binding proteins (RBPs) in human [3, 12–17], while an additional 1500 RBPs have been computationally predicted [18]. Moreover, cross-linking/immunoprecipitation with sequencing (CLIP-seq) experiments have shown that a typical RBP interacts with hundreds to thousands of different RNAs [19–23]. Conversely, some RNAs are targeted by a handful of RBPs, while others bind to hundreds of them.

Importantly, recognition between the known RNA-binding domains (RBDs) and their target motifs only partly explains the large-scale organization of RNA–protein interaction networks. For example, approximately one half of all known RBPs in human do not contain any known RBDs and many are heavily structurally disordered [3, 10–13, 24]. Moreover, slight variations of the consensus RBD sequence can greatly alter binding mechanism and target specificity [25, 26], while distinct RBDs can modularly combine to yield varied outcomes [27–29]. This gap in understanding clearly prompts the need for new explanatory frameworks [30]. Going beyond individual binary interactions, what appears to be particularly missing is a clear understanding of the general principles that define the large-scale architecture of RNA–protein interaction networks. Specifically, little is known about the connection between the general properties of individual target RNAs and the collective properties of the RBPs that interact with them.

Growing evidence suggests that bulk compositional features, going beyond local sequence motifs, also influence RNA–protein recognition. Multiple RBPs preferentially bind RNAs enriched in certain nucleotides, such as TIA-1 (URA-rich) [31], PCBPs (CYT-rich) [32], PABPC1 (ADE-rich) [33], and FUS (GUA-rich) [34], or dinucleotides, especially GUA/CYT or ADE/URA [35, 36]. These compositional biases align with broader structural and functional nuclear organization, particularly when it comes to the formation of biomolecular condensates [37]. Indeed, the accumulation of condensation-prone, arginine-rich proteins in nuclear speckles has recently been shown to drive “interstasis,” a specific homeostatic feedback loop that selectively sequesters their own purine-rich mRNAs to manage protein dosage [38]. Moreover, recent studies reveal that the human genome is spatially organized along a gradient in composition extending from the lamina to nuclear speckles [39–41]. In particular, chromatin domains surrounding the speckles are notably enriched in GUA/CYT-rich sequences and actively transcribed genes [39–41], while speckle-localized pre-mRNAs characteristically have short introns and high GUA/CYT content [42, 43]. These sequence-based gradients are mirrored at the protein level, as canonical speckle-associated proteins possess low-complexity, arginine- and serine-rich (RS) domains promoting liquid–liquid phase separation and RNA binding [44–46].

Given the high degree of structural disorder of RBPs [24], the intrinsic affinities between amino acids and nucleotides [47–49] do play a significant role in establishing RNA–protein binding specificity [50, 51]. Related to this, there is evidence that amino acids may preferentially interact with the nucleotides that feature in their own codons, reflecting the driving forces behind the origin of the genetic code [47, 51–53]. It was suggested that this may establish a link between mRNAs and the proteins they encode, going beyond their coding relationship [50, 51, 54]. In particular, it was proposed that mRNAs and their autogenous proteins may in general exhibit affinity for each other and bind in a co-aligned manner, especially if unstructured [49–51, 54, 55]. In support, it was shown that approximately two-thirds of all RBPs ever studied by crosslinking and immunoprecipitation with sequencing (CLIP-seq) bind directly to their own autogenous mRNAs [56].

Importantly, it was suggested that proteins may also preferentially interact with non-autogenous mRNAs that are compositionally related to their own autogenous mRNAs and vice versa: a given mRNA may prefer to interact with a set of RBPs with whose mRNAs it is compositionally related [51, 56]. If genetic coding in part evolved from RNA–protein interactions, then RNA–protein interactions may be rationalized from the perspective of coding and the relationships it implies. Here, we systematically test this hypothesis using eCLIP data for 150 human RBPs [4, 22, 57]. We represent each protein by the nucleotide composition of its full spliced transcript (coding sequence, CDS + untranslated regions, UTRs) and show that, across the transcriptome and for all four nucleotides, mRNAs preferentially bind RBPs whose own transcripts mirror their own compositional bias. The trend persists in a cell-free setting, as confirmed with HTR-SELEX data [58], and extends to nuclear organization. By integrating proximity-labeling data [40, 42], we link mRNA speckle association with their compositional binding preferences and to colocalization to their autogenous proteins. Our results uncover a robust, compositionally rooted layer of organization behind RNA-protein interactions, which directly links the structure of the genetic code with the spatial and functional architecture of gene regulation.

## Materials and methods

### CLIP-seq data selection and acquisition

Protocol heterogeneity in CLIP can introduce artifactual nucleotide- or sensitivity biases [4, 59], which could obscure genuine binding patterns in the context of cross-protein comparisons. To ensure maximal consistency, we focused on enhanced CLIP (eCLIP) datasets generated by the ENCODE Consortium [4, 22, 57], which capture interactions of 150 human RBPs profiled in HepG2 and K562 cells, all produced with an identical laboratory workflow and processed through a single peak-calling pipeline. Processed peak data were obtained through POSTAR3 [23] and for each RBP, peaks from biological replicates were merged.

### Transcript data acquisition and selection

We mapped eCLIP reads to canonical transcript variants defined by the MANE [60 ] database (v1.2), which provides single, high-confidence transcripts for over 19 000 human genes. For transcripts not included in MANE (e.g. NKRF), the highest-confidence transcript was selected based on ENSEMBL BioMart [61] annotations. In all analyses, we studied full, mature mRNA transcripts (CDS + UTRs), if not indicated otherwise. Introns were excluded to focus on spliced transcripts and their compositional effects. MANE data were downloaded from https://ftp.ncbi.nlm.nih.gov/refseq/MANE/, and supplementary transcript information was retrieved from ENSEMBL BioMart: https://www.ensembl.org/biomart/martview.

### Definition of mRNA–protein interactions

For eCLIP data, any transcript with at least one cross-linking peak called at default thresholds was regarded as interacting with the RBP in question. For HTR-SELEX data, each transcript with the RBP’s primary and secondary motifs was scanned and sites exceeding a score threshold corresponding to *P *≤ 0.0001 identified. For motifs up to 16 nucleotides in width the thresholds were determined via an exact method under a second-order Markov chain model, whereas thresholds for wider motifs were estimated on the basis of random sampling (*N* = 10/*p* trials).

### Calculation of Interactome Codedness Preference and feature preferences

We introduced the Interactome Codedness Preference (ICP) metric to quantify compositional biases in the encoding transcripts of RBPs interacting with a given mRNA. For each mRNA, the mean nucleotide content (G, A, C, U) of the encoding transcripts of its interacting RBPs was calculated. This observed average was then compared to a simulated background distribution. Specifically, for each mRNA, we drew random subsets of RBPs of the same size from the global RBP pool (i.e. the 150 eCLIP or 42 HTR-SELEX RBPs) without replacement. To ensure that each RBP’s overall interaction frequency was preserved, the probability of selecting a specific RBP during this randomization was strictly proportional to its total number of target interactions in the dataset. By repeating these probability-weighted random draws 10 000 times, we built a null distribution of average transcript nucleotide contents. The deviation of the actual observed average from this null distribution was finally expressed as a *z*-score, defined as the ICP value of the mRNA in question. ICP was calculated independently for ADE, CYT, GUA, and URA. A Python script demonstrating the core algorithmic processing is provided as Supplementary Data S8. This identical statistical framework was also employed to calculate analogous binding preferences for other RBP features, substituting the RBP transcript nucleotide content with alternative quantitative or binary features, such as the mean amino acid GUA-affinity of the RBP or its speckle-association status, as demonstrated in Supplementary Data S8.

### STREME motif discovery in eCLIP data

To identify high-confidence binding sites and mitigate transcript abundance biases, we filtered binding sites for replicate agreement. Specifically, within each genomic transcript span, we identified the position with the highest count of mapped peaks, using peak-intersection length as a tiebreaker. These sites were centered and extended by 50 nucleotides (nt) in both directions according to the GRCh38 reference genome, yielding 101-nt window sequences. Motif discovery was then performed on these sequences using STREME [62] with RNA-specific parameters (–rna), a motif width of 6 to 15 nt (–minw 6 –maxw 15), and a shuffled input-sequence background (default). Crucially, correcting for monomer composition isolates sequence-specific from composition-specific binding, which we investigate in the present analysis. Applying this pipeline to 150 eCLIP RBPs with a lenient *E*-value cutoff of < 0.2 (default: 0.05) identified motifs for 126 RBPs, yielding an average of 2.74 motifs per RBP with a mean length of 11.1 nt.

### Removal of motif-intersecting eCLIP-binding sites

As a control experiment, we removed binding sites that could be explained by linear motif sequences. First, all overlapping peaks from replicates were merged, resulting in 624 479 unified peaks across the 150 RBPs that mapped to MANE transcripts. Next, we excluded any unified peaks that at least partially overlapped with their respective RBP-binding motifs. Specifically, the STREME motifs identified above were scanned using a lenient *P*-value cutoff of 0.001 (default: 10^-4^). The extent of peak overlap varied significantly by RBP, reaching up to 89.4% for HNRNPC. Overall, this filtering step resulted in the removal of 42.3% of all unified peaks and 31.4% of the total RNA–protein interactions. When using MEME’s default motif discovery and allocation cutoffs (*E *< 0.05; *P* < 10^-4^), this process removes 8.96% of all unified peaks.

### HTR-SELEX data acquisition

*In vitro* data generated by High-Throughput RNA SELEX (HTR-SELEX) containing motif information for 86 human RBPs *(“PWMs-linear”)* was retrieved from Jolma *et al*. [58]. As approximately one half of these RBPs were studied as truncated constructs only, which is incompatible with the scope of the present analysis of compositional features of full mRNAs, we focused on the 42 proteins present in full-length form. Both primary and secondary motifs, as captured by the respective PMWs, were considered for each RBP.

### Protein sequences and GUA-affinity scaling

Amino-acid sequences for the investigated RBPs and background nuclear proteins were extracted from the MANE database (v1.2, MANE.GRCh38.v1.2.ensembl_protein.faa.gz). To calculate a theoretical mean GUA-affinity per protein, we utilized published nucleobase-affinity scales derived from bead-based fluorescence assays [63] and molecular dynamics simulations [64]. For each protein, the mean GUA-affinity was computed as a weighted average: the fractional frequency of each of the 20 standard amino acids in the protein sequence was multiplied by its corresponding GUA-interaction energy from the respective scale, and these values were summed across the entire protein.

### Speckle association data acquisition

We integrated three complementary datasets to evaluate the proximity of transcripts, chromatin and proteins to nuclear speckles, mapping all entries to single MANE (v1.2) transcripts where possible. First, RNA-domain association data, as given in [42], identified genes enriched in various nuclear compartments, including speckles. Second, TSA-seq measurements, as given in [40], provided percentile-based rankings of chromatin positions relative to speckles; these values served to capture a continuous range going from lamina to speckle. Finally, the data from [65] were used to capture protein-level speckle enrichment via a speckle-to-centromere ratio.

### Covariate-controlled analyses

To decouple the observed compositional trends from potential systemic confounders, we performed covariate-controlled analyses on restricted subsets of mRNAs. Subsets were generated by taking 1000 values (also including ties) around the global median for three key variables: transcript length, expression level, and the total number of bound RBPs. These selection windows were significantly narrower than the overall transcriptome distribution to ensure the removal of systemic bias. For transcript length, this yielded 1002 transcripts with lengths between 3125 and 3623 nucleotides, compared to global 10th–90th percentiles of 1326–7708 and a median of 3374 nt. For expression level (Transcript per million, TPM), this yielded 1008 transcripts with expression levels between 6.25 and 8.95 TPM, compared to global 10th–90th percentiles of 0.10–54.32 and a median of 7.6 TPM. For interaction frequency, this yielded 1344 transcripts with interaction frequencies between 14 and 18 bound RBPs, compared to global 10th–90th percentiles of 2–42 and a median of 16 bound RBPs. TPM values were obtained from the Human Protein Atlas (HPA) rna_celline.tsv dataset, using the mean TPM across HepG2 and K562 cell lines to match the eCLIP cellular context, while transcript length was defined by the canonical MANE Select transcript.

### Non-coding RNA analysis

To investigate compositional binding patterns in non-coding transcripts (lncRNAs), eCLIP peaks from POSTAR3 were mapped to human long intergenic non-coding RNAs (lincRNAs) and antisense RNAs. Transcript coordinates and sequences were defined using GENCODE v49 annotations (.gtf and .fa) alongside the GRCh38 reference genome. Transcripts were considered interacting if an eCLIP peak mapped within their boundaries. Unlike mRNAs, which are accurately represented by a stable, fully spliced dominant isoform (e.g. MANE Select), lncRNAs are inefficiently spliced and exhibit a higher transcript complexity, yielding numerous low-abundance splice variants rather than a single definitive sequence [66]. With inadequate knowledge of the full-polymer sequence, lncRNA nucleotide compositions were instead represented by their experimentally measured eCLIP-binding sequences. ICP values for lncRNAs were then calculated using the identical probability-weighted sampling framework applied to mRNAs.

### Statistical analyses and bootstrapping

Data processing and statistical analyses were performed using Python 3.11, utilizing NumPy (v1.26.2) and Pandas (v2.1.4) for data manipulation, and SciPy (v1.11.4) for statistical testing. Mann–Whitney *U* tests were computed using scipy.stats.mannwhitneyu with default parameters (two-sided), unless evaluating the directional reproduction of previously established trends, in which case one-sided tests were applied as explicitly noted in the text. To assess the robustness of the compositional correlations between mRNA nucleotide contents and their ICP values, bootstrap analyses were conducted. We systematically removed varying numbers of RBPs from the eCLIP dataset (*N* = 1, 2, 3, 5, 10, 20, 40, 60, 100) at random and recalculated the ICP values for all mRNAs. For each *N* ≥ 2, the removal and recalculation process was repeated 10 000 times. For *N* = 1, each of the 150 RBPs was systematically removed one at a time to analyze individual impact. Finally, to evaluate whether the global auto-compositional binding pattern is driven solely by a few RBPs with extreme compositional biases, we performed a symmetric robustness test by systematically removing RBPs pairwise from both ends of the impact spectrum (i.e. simultaneously removing the most significant positive and negative contributors) and re-evaluating the statistical separation.

## Results

### mRNA interactomes exhibit distinct encoding patterns

**Figure 1.**
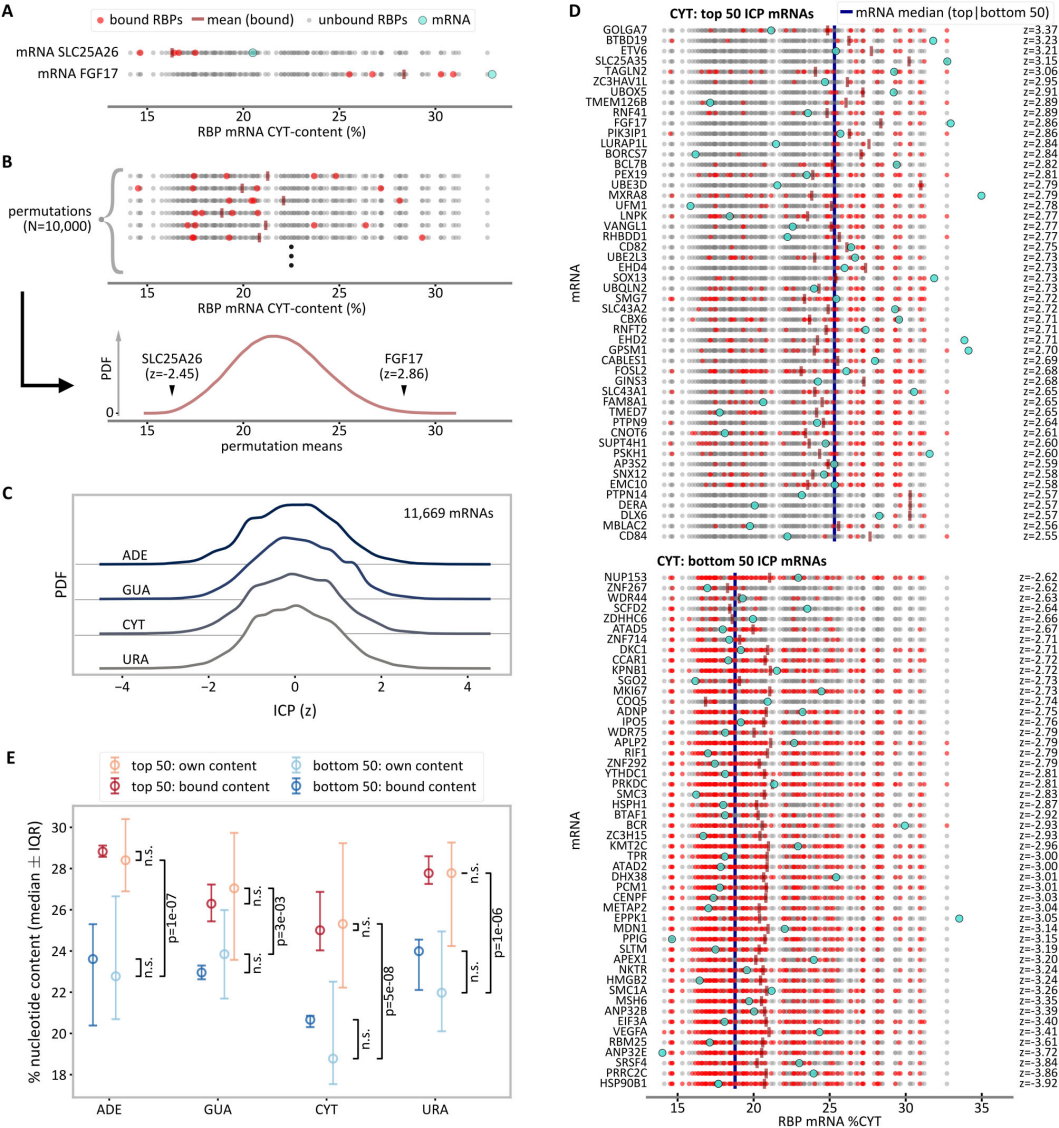
mRNAs are regulated by compositionally distinct sets of RBPs. (**A**) mRNA CYT-content of the four eCLIP RBPs that bind to SLC25A26 and FGF17 mRNAs (red dots, with the average CYT-content depicted with a red bar), together with the mRNA CYT-content of the eCLIP RBPs that do not bind to these mRNAs (gray dots). The CYT-content of SLC25A26 and FGF17 mRNAs themselves is indicated using cyan dots. (**B**) workflow for ICP calculation: ICP is the *z*-score of the average mRNA nucleotide content of the transcripts of the RBPs that interact with a given mRNA as compared to the distribution of the average RBP mRNA nucleotide contents for 10 000 randomized permutants of equal size. The actual ICP_CYT_ values for SLC25A26 and FGF17 are indicated by arrows. See Materials and methods for details. (**C**) Distributions of ICP values for URA, CYT, ADE, and GUA over all eCLIP mRNAs. (**D**) Encoding mRNA CYT-contents (red dots) of the RBP interactomes of the top and the bottom 50 target mRNAs when it comes to their ICP_CYT_ values. For a given target mRNA, the average CYT-content of its interacting RBP mRNAs is depicted with red bars, while its own CYT-content is given as a cyan dot. The average CYT-content of the two target mRNA groups are indicated as blue vertical lines. (**E**) Median nucleotide content (±IQR) for the top and bottom 50 ICP mRNAs and their interacting RBPs’ mRNAs across different nucleotides (ADE, GUA, CYT, and URA). The plot compares the top 50 ICP mRNAs (salmon) against the bottom 50 (light blue), with *P* < 0.01 for all nucleotides (Mann–Whitney *U*; two-sided). In contrast, mRNA’s own nucleotide-content (salmon/light blue) generally matches that of bound RBP’s mRNAs (red/ dark blue), showing insignificant (n.s.) differences in medians (*P* ≥ 0.01) in all cases.

From the perspective of an individual mRNA, is its nucleotide composition related to how the proteins it binds are encoded? If the intrinsic affinities between different nucleotides and amino acids indeed define codon assignment [47, 51–53], they may likewise promote *in vivo* interactions between mRNAs and RBPs whose own transcript composition mimics that of the mRNAs. We illustrate this concept on the example of the cytosine (CYT) content of human mRNAs SLC25A26 and FGF17, which both interact with exactly four RBPs from the eCLIP [4, 22, 57] dataset (Fig. 1A). The RBPs interacting with SLC25A26 mRNA are, on average, encoded by CYT-poor mRNAs (<CYT ≥ 17%), while those interacting with FGF17 mRNA are, on average, encoded by CYT-rich mRNAs (<CYT ≥ 28%) (Fig. 1A). Intriguingly, the SLC25A26 mRNA itself also exhibits a significantly lower CYT-content as compared to the FGF17 mRNA (20.5% versus 33.0%, respectively) (Fig. 1A).

How does this singular observation translate to the full human transcriptome? To quantify such trends while accounting for the fact that different mRNAs and RBPs interact with different numbers of partners, we define an mRNA’s ICP as the *z*-score of the average nucleotide content of its partner RBPs’ mRNAs as compared to the background distribution obtained for randomized, interaction-frequency-weighted permutants (Fig. 1B, see Materials and methods for more details). As expected, the ICP_CYT_ of SLC25A26 mRNA is significantly lower than that of FGF17 (−2.45 versus 2.86, Fig. 1B). In fact, these two mRNAs were chosen as examples because they exhibit the lowest and the highest ICP values, respectively, among all human mRNAs that bind exactly four RBPs. Overall, the genome-wide ICP values are approximately normally distributed and span >8 standard deviations for each nucleotide (Fig. 1C). Clearly, different mRNAs are bound by distinct sets of RBPs when it comes to the average composition of their own encoding transcripts.

### mRNA composition mirrors how its bound proteome is encoded

ICP now allows us to study at the level of the complete cellular interaction network the relationship between the composition of target mRNAs and the mRNAs that encode their interacting RBPs. In Fig. 1D, we first focus on the extremes of the ICP distribution and present the complete interactomes of the top and the bottom 50 human mRNAs when it comes to ICP_CYT_ values. It is visually immediately apparent that mRNAs with the highest ICP values i.e. those that interact with RBPs whose own transcripts are most significantly CYT-enriched, exhibit a median CYT-content that is significantly higher than that of the mRNAs with the lowest ICP values (Fig. 1D; 28.9% versus 22.8%, respectively; *P *< 10^−7^, Mann–Whitney *U*). A similar pattern is seen for the other three RNA nucleotides as well (Fig. 1E, and Supplementary Figs S1 and S2). Moreover, for every nucleotide type and top/bottom category, the median mRNA composition matches that of their bound RBPs’ mRNAs (e.g. CYT 28.6 % versus 28.9 %; *P* = 0.56 (n.s.), Mann–Whitney *U*, Fig. 1E).

**Figure 2.**
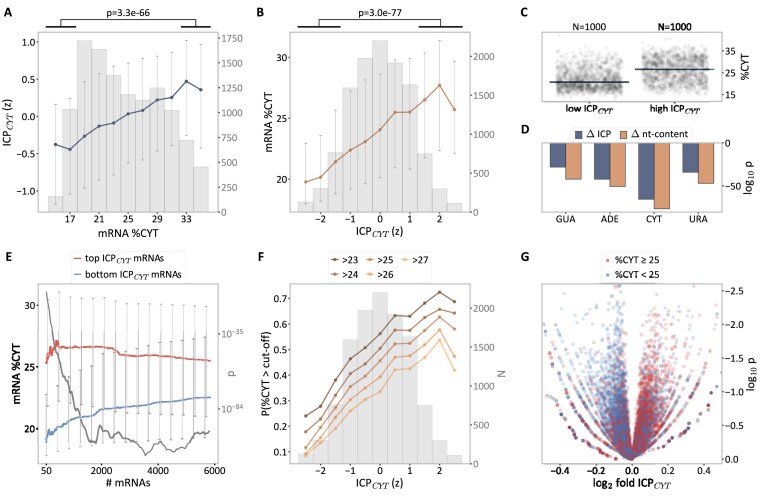
mRNA nucleotide content is related to its ICP. (**A**) Histogram of target mRNA CYT- content (*N* = 11 669; bin-width = 2%; right-hand *y*-axis) and the corresponding bin-median ICP_CYT_ values (left-hand *y*-axis). (**B**) Histogram of mRNA ICP_CYT_ (*N* = 11 669, bin-width = 0.5, right-hand *y*-axis) and the corresponding bin-median mRNA CYT-content (left-hand *y*-axis). Error bars represent the associated interquartile ranges. The *P*-values above the panels (A) and (B) refer to the statistical significance of the difference in the *y*-axis variable between the sets of the top and the bottom 1000 mRNAs, as ranked according to the variable on the *x*-axis (Mann–Whitney *U* test). The corresponding *x*-axis ranges are indicated by the horizontal lines above. (**C**) Jitter plot of CYT-content of the top and the bottom 1000 mRNAs according to ICP_CYT_. (**D**) log_10_  *P*-values, as shown in panels (A) and (B), for all 4 nucleotides. (**E**) Median mRNA CYT-content of the *N* mRNAs with the highest (red) and the lowest (blue) ICP_CYT_ as a function of *N*. In panels (E) and (F), the error bars capture the interquartile ranges, while the associated *P*-values refer to the difference between the two sets at a given *N* as evaluated by Mann–Whitney *U* test. (**F**) Histogram of mRNA ICP_CYT_ (*N* = 11 669, bin-widths = 0.5, right-hand *y*-axis) and the corresponding fraction of mRNAs in a given bin that exceed a minimum CYT-content cutoff, as indicated above the panels (lines, left-hand *y*-axis). (**G**) Volcano plot representing 11 669 mRNAs and the RBP sets that target them. The *x*-axis shows the log_2_ enrichment of the CYT-content of the interacting RBP for each target mRNA, while the *y*-axis indicates the log10 *P*-value of the enrichment. Red points indicate CYT-rich target mRNAs (%CYT ≥ 25), while blue dots indicate CYT-poor target mRNAs (%CYT < 25). Dots are displayed with 80% transparency and randomized *z*-stacking.

The above relationship remains robust and statistically significant across the full range of mRNA compositions in the expressed transcriptome (*N* = 11 669) for all four RNA nucleotides, with the average ICP increasing almost linearly with an increase in the average mRNA nucleotide content (Fig. 2A and Supplementary Fig. S3A). Notably, the 150 autogenous mRNAs of the eCLIP RBPs are among this set and could theoretically slightly promote the observed trend, given that they generally tend to bind their own RBPs [56]. On the other hand, these cases represent only 150 out of over 1.75 million possible mRNA-protein interactions in the studied set and are not expected to affect any global trends. Indeed, a repeated analysis after omitting these 150 mRNAs led to nearly indistinguishable results (data not shown). Similarly, and statistically even more significantly, nucleotide content rises in proportion to ICP (Fig. 2B and Supplementary Fig. S3B). These global trends are particularly apparent at the extremes of the distribution. For instance, the average CYT-content of the 1000 transcripts with the highest ICP_CYT_ values is 26.4%, to be compared with 21.9% for the 1000 transcripts with the lowest ICP_CYT_ values (Fig. 2B and C; *P*-value < 10^−76^, Mann–Whitney *U* test). For an mRNA with 2942 nucleotides (median length in human), this difference in the average CYT-content translates into an additional 132 CYT nucleotides in mRNAs that interact with RBPs whose own mRNAs are CYT rich as compared to those that interact with RBPs whose own mRNAs are CYT poor. Similar differences are seen for all four RNA nucleotides (Fig. 2D). Furthermore, one sees an analogous pattern in the reverse case as well, comparing the average ICP values between the top and the bottom 1000 mRNAs by nucleotide content, for all four standard RNA nucleotides (Fig. 2D). Also, varying the number N of the top and the bottom mRNAs by either nucleotide content (Supplementary Fig. S4A) or ICP (Fig. 2E and Supplementary Fig. S4B) in these comparisons results in extremely statistically significant differences across the full range of *N* going from 50 to ∼6 000 mRNAs for all four nucleotides. Finally, all of the observed patterns persist whether analyzing full mRNA and RBP transcript sequences, their CDS, or combinations thereof, except for uracil (data not shown).

Analysis of the probability that ICP exceeds a given threshold as a function of mRNA nucleotide content (Supplementary Fig. S5A) provides a more nuanced understanding of these trends. For example, the probability that ICP_CYT_ > 0 doubles going from mRNAs with a CYT content <16% to those with a CYT content > 34% (0.32 versus 0.63, respectively ), with systematically increasing values in the intermediate range. Similar trends are observed for all other ICP cutoffs, ranging from −1 to 1, and for all nucleotides (Supplementary Fig. S5A). The probability that mRNA nucleotide content exceeds a given threshold as a function of its ICP is even more striking (Fig. 2F and Supplementary Fig. S5B). The probability that an mRNA’s CYT content is >25% increases >4.6-fold going from mRNAs with an ICP_CYT_ < −2 to those with an ICP_CYT_ > 2 (0.13 versus 0.62 ), with systematically rising values in between.

To address whether these correspondences might arise from shared expression contexts or systemic biases rather than intrinsic biochemical affinity, we performed a covariate-controlled analysis by selecting restricted subsets of transcripts (Supplementary Fig. S6). As detailed in the Materials and methods section, the variables for cDNA length, expression (TPM), and interactivity were confined tightly around their global medians, utilizing a selection range width approximately between 10- and 20-fold narrower than the 10th–90th percentile range of the full distribution. Even when restricted to approximately 1000 mRNAs (compared to the original 11 669) and testing differences between the top and bottom 100 mRNAs (instead of 1000), the statistical significance remained remarkably high, with *P*-values consistently between *P* < 10^−4^ and *P* < 10^−16^ (Supplementary Fig. S6). Notably, these constraints appeared to reduce statistical noise, leading to effect sizes that were often larger than those observed in the global analysis; for instance, in the length- and expression-controlled subsets, the average CYT content differs by over 10 percentage points between the lowest and highest ICP_CYT_ bins, rising from ∼18% to 28%, a span wider than that seen in the full transcriptome (∼19%–27%). These observations are consistent with global analyses of the full transcriptome, which indicate that mRNA interaction frequency is not a major confounding factor (Supplementary Fig. S7). While GUA- and ADE-rich mRNAs tend to bind slightly more RBPs, CYT- and URA-contents show no meaningful correlation with RBP-binding counts. This reflects the biological tendency of high GUA- and ADE-encoded proteins to bind more transcripts (data not shown), rather than a mathematical artifact where high or low binding numbers automatically lead to specific ICP values.

Crucially, to distinguish whether these genome-wide trends are driven solely by specific, high-affinity RNA recognition motifs or reflect a broader mode of interaction, we repeated the analysis after filtering out eCLIP binding sites overlapping with discovered linear sequence motifs (see Materials and methods). In the original analysis, these global correlations were extremely robust (*P*-values between 10^-77^ and 10⁻²⁸; Supplementary Fig. S3). Applying a standard motif discovery and removal threshold using MEME’s default cutoffs (*E* < 0.05, *P* < 10⁻^4^) removed 8.96% of all unified peaks and yielded only slight statistical changes to the correlations (*P*-values between 10⁻^70^ and 10⁻^24^; Supplementary Fig. S8). To rigorously test the limits of this observation, we subsequently removed all eCLIP-binding sites overlapping with leniently discovered sequence motifs (*E* < 0.2, *P* < 0.001). This strict procedure removed over 80% of binding sites and mRNA targets for strongly motif-driven RBPs like HNRNPC, HNRNPK, QKI, and TARDBP, and 42.3% of binding sites across all 150 RBPs overall. While the statistical significance is lower than in the primary analysis under this stringent removal—which eliminates not just exact motif sequences but binding sites that loosely resemble them—the global correlations between mRNA nucleotide content and ICP remain qualitatively unchanged and highly significant for all four nucleotides (*P*-values between 10⁻^41^ and 10⁻^9^; Supplementary Fig. S9). The persistence of this signal in the strict absence of motif-driven interactions suggests that the observed compositional matching does not stem from a coincidental alignment of RBPs’ sequence motifs with their own transcript contents. Instead, it is consistent with the idea of underlying physicochemical attractions between transcript and peptide bulk compositions. Finally, a volcano plot analysis underscores the fact that positive ICP shifts align with nucleotide enrichment and vice versa (Fig. 2G).

To decouple RNA sequence composition from the evolutionary biases that shape functionally related, co-expressed gene clusters, we investigated long non-coding RNAs (lncRNAs). Within this context, we also specifically evaluated antisense RNAs to test if local DNA spatial organization or locus-associated processing machinery drives the patterns; originating from the opposite strand, their complementary compositions should theoretically yield reversed correlations. Initially, evaluating antisense RNAs and long intergenic non-coding RNAs (lincRNAs) independently yielded no significant patterns (*P *> 0.05). This lack of signal is partly due to sparse detection in the eCLIP dataset, consistent with the characteristic low abundance of lncRNA transcripts [66]: only 1098 antisense RNAs and 1085 lincRNAs bind at least one RBP. More critically, this limitation is compounded by a median of just two bound RBPs per lncRNA, compared to 16 for mRNAs. Because robust binding preferences are established by comparing an aggregate of interactions against a background distribution, evaluating transcripts with only one or two bound RBPs fails to capture meaningful deviations, yielding scores that are dominated by statistical noise. However, upon combining these subgroups into a broader lncRNA dataset (*N* = 2183), the patterns observed in mRNAs were partially reproduced in the same directionality (not reversed). Despite the inherent statistical noise, this effect remains significant for cytosine (*P *> 0.05 for other bases), the base exhibiting the strongest trends in mRNAs. Specifically, when comparing the top and bottom 1000 lncRNAs, those that bind high-cytosine-coded RBPs are themselves enriched in cytosine within their binding regions (*P *= 0.009, Mann–Whitney *U* test, two-sided), and vice versa (*P *= 0.004). To study the impact of lncRNAs with poorly defined eCLIP-binding profiles, we have grouped together all lncRNA that have at least a given number of RBPs bound, with the cutoff rising incrementally from 1 to 30 (Supplementary Fig. S10). At each cutoff, transcripts were bisected into equal cohorts (top versus bottom 50%) based on nucleotide content (Supplementary Fig. S10A) or ICP score (Supplementary Fig. S10B). As the number of bound RBPs increases, the respective spread between cohorts generally widens for all four bases. In particular, at an intermediate threshold of ≥16 bound RBPs (*N* = 301), stratifying lncRNAs by CYT peak sequence content shifts the median ICP_CYT_ from −0.42 in the bottom 50% to 0.15 in the top 50% (*P *= 1.3 × 10^−06^; Supplementary Fig. S10A). Conversely, stratifying by ICP_CYT_ yields a robust median peak content gap of 22.1% versus 25.6% (*P *= 2.4 × 10^−05^; Supplementary Fig. S10B). Eventually, for very high cutoffs, the significance reduces most likely due to the loss of sample size. Repeating the equivalent analysis representing lncRNAs by their full, unspliced sequences, instead yields *P*-values of 5.8e-03 and 6.9e-04, respectively. Finally, the analysis results in qualitatively similar patterns for all other nucleotides, but with statistical significance (*P *< 0.05) being reached at certain cutoffs by URA only (Supplementary Fig. S10).

### Large-scale organization of the mRNA–RBP interaction network

**Figure 3.**
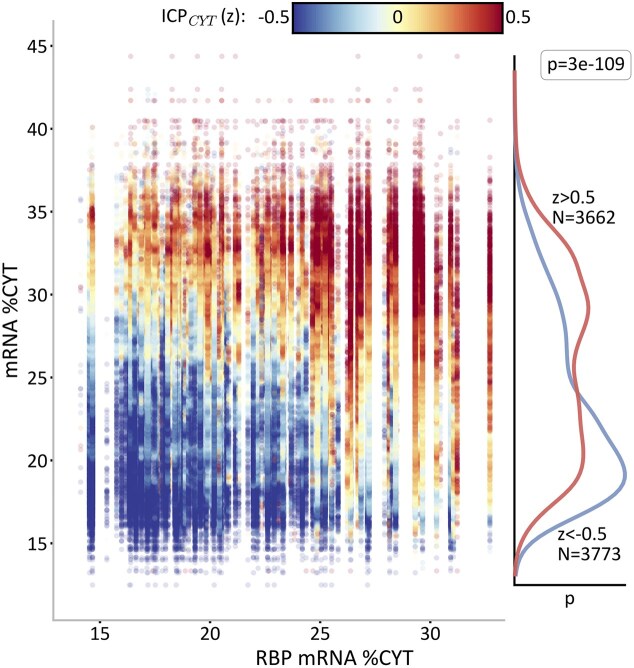
Compositional gradients dominate the large-scale organization of the human RNA–protein interaction network. eCLIP RNA–protein interactome (150 RBPs versus 11 669 mRNAs), as represented by RBP mRNA and target mRNA CYT-content. Dots corresponding to the RBPs interacting with a given mRNA (horizontal lines) are colored according to this mRNA’s ICP_CYT_ (legend given above the graphs). Overlapping dots are shown in the same color, representing the mean of nearby dots within d*x* = 0% and d*y* ≤ 0.5%. Distributions of %CYT of mRNAs with ICP_CYT_ > 0.5 (red) and < -0.5 (blue) with the associated *P*-values (Mann–Whitney *U*, two-sided) are given on the right. The number of mRNAs behind each distribution is given next to the curves.

The most comprehensive and unfiltered view of the above dependencies is obtained by representing the complete eCLIP mRNA–RBP interaction network point-by-point using RBP transcript nucleotide content and target mRNA nucleotide content as coordinates. Each detected interaction is represented by a dot and colored according to the mRNA’s ICP value for CYT (Fig. 3) or one of the other nucleotides (Supplementary Fig. S11). Overlapping dots are given the same color, representing the mean of nearby dots within d*x* = 0% and d*y *≤ 0.5%. As an alternative visualization that bypasses the need for color-averaging of multiple mRNAs, Supplementary Fig. S12 shows an equivalent plot that focuses on mRNA–protein interactions involving the top 1000 (colored red) and bottom 1000 mRNAs per ICP (colored blue).

Expectedly, interactions involving mRNAs with low ICP values for a given nucleotide (blue dots) group on the left-hand side, while those with high ICP values (red dots) group on the right-hand side. This pattern along the *x*-axis is expected given the definition of ICP. Remarkably, these graphs also reveal a striking continuous pattern along the *y*-axis: high ICP values for a given nucleotide predominate at the top (high target mRNA nucleotide density), while low ICP values predominate at the bottom (low target mRNA nucleotide density), with a smooth gradient in between, as shown for CYT (Fig. 3) and other nucleotides (Supplementary Fig. S11). To provide a more quantitative perspective, in Fig. 3 (CYT) and Supplementary Fig. S11 (all nucleotides), we also show the complete probability densities of the nucleotide contents of mRNAs with ICP > 0.5 (red) and ICP < -0.5 (blue), corresponding to 7435 of the 11 669 mRNAs. For each nucleotide, the distributions are strongly separated, with associated *P*-values < 10^−65^ (Mann–Whitney *U* test, two-sided).

Interestingly, there appears to be a notable difference between ADE and URA on the one hand and GUA and CYT on the other, regarding what creates the bulk of the difference between mRNAs with an ICP > 0.5 and those with an ICP < -0.5 (Supplementary Fig. S11). For ADE and URA, mRNAs with an ICP < -0.5 do not show a marked preference in content, while those with an ICP > 0.5 are highly enriched in ADE or URA. For GUA and CYT, it is the mRNAs with an ICP < -0.5 that show a strong preference, being depleted in GUA and CYT, respectively, while those with an ICP > 0.5 are approximately equally distributed between low and high GUA- and CYT-content. These findings suggest mechanistic idiosyncrasies that should be further investigated in the future.

### Multiple RBPs contribute synergistically to compositional binding patterns

Could the above genome-wide trends be caused by just a few particular RBPs? To analyze this, we have first ranked all 150 eCLIP proteins by (i) the raw number of interactions they make with the 1000 transcripts with the highest ICP_CYT_ values, and (ii) their enrichment in that subset relative to the full network (Supplementary Fig. S13). No single protein dominates: the proteins with the longest partner lists show only modest selectivity (for half of the top ten the enrichment above baseline is <20 %), whereas the proteins with the strongest enrichment tend to interact with few targets (five of the top ten bind fewer than 50 transcripts). Thus, individual RBPs contribute either a broad but shallow or a narrow but deep bias, while none exert both forms of influence simultaneously.

**Figure 4.**
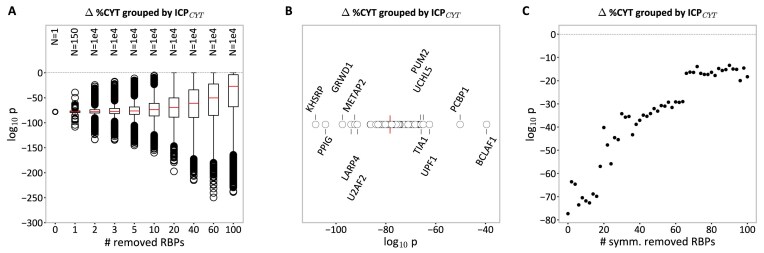
Bootstrapping reveals robustness of the auto-binding relationship. (**A**) log10 *P*-values (one-sided) obtained after removing 0–100 out of 150 eCLIP RBPs and evaluating the statistical significance of the separation in %CYT between the top and the bottom 1000 mRNAs as ranked by ICP_CYT_. The *x*-axis indicates the number of RBPs removed. Red lines represent the median *P*-values of *N* randomization trails. (**B**) Equivalent to panel (A), but showing the effect of removing each of the 150 eCLIP RBPs individually. Red line indicates the starting *P*-value i.e. when all 150 RBPs are included. The six RBPs with the highest and the lowest *P*-values are annotated explicitly. (**C**) Similar to panel (A), but with RBP removed pairwise based on their impact, as determined in panel (B): for each *N*, the leftmost and the rightmost outlier RBPs were removed sequentially e.g. *N* = 20 represents removal of the ten most extreme RBPs on both sides.

Robustness bootstrap analysis reinforces this view. When random subsets of RBPs were removed and the difference in composition between 1000 transcripts with the highest and 1000 with the lowest ICP_CYT_ values reevaluated, a loss of up to 20 proteins left the median *P*-value virtually unchanged. Even deleting two-thirds of the dataset (100/150 RBPs) preserved a highly significant difference (median *P* ≈ 10^-27^, Mann–Whitney *U*; Fig. 4A). Only when rare combinations of multiple RBPs were removed (<0.1% of iterations) did we observe a significant reduction in the observed correlations (Fig. 4A), implying that the trends should remain stable as additional RBPs are characterized. Equivalent analyses for ADE, GUA, and URA support the same conclusion (Supplementary Fig. S14).

Finally, we systematically removed each RBP, one at a time, and examined the impact on this analysis (Fig. 4B and Supplementary Data S7). Eliminating BCLAF1 or PCBP1 led to the steepest loss of significance, consistent with their extreme compositional biases: BCLAF1 is encoded by a CYT-poor transcript (16.6 %) and systematically avoids CYT-rich mRNAs (the average composition of its partners being 51.6 standard deviations below a size-matched random expectation), whereas PCBP1, a canonical poly-C binder [32], is encoded by a CYT-rich transcript (29.5 %) and preferentially targets CYT-rich RNAs (Fig. 4B). Conversely, removal of KHSRP enhanced the trend: although its own transcript is CYT-rich (31%), the protein itself favors CYT-poor targets (∼33.5 standard deviations below random).

The extreme binding specificities of certain RBPs, such as BCLAF1, PCBP1, or KHSRP, may be a consequence of specific, evolutionarily optimized functional constraints that are independent from the above trends. In Fig. 4C, we demonstrate that the global pattern remains robust even after symmetrically removing up to 100 RBPs pairwise from both ends of the spectrum based on their impact as evaluated above. Despite removing the most significant contributors, the overall signal persists, indicating that the compositional binding trends are not engendered by these extreme cases only (Fig. 4C). This suggests that, while certain RBPs do exert a disproportionate influence, the general pattern of auto-compositional binding, i.e. that RBPs’ own transcripts tend to reflect composition of target mRNAs, remains a fundamental feature of RNA–protein interactions for most RBPs.

### Compositional binding biases persist *in vitro*

A natural question is whether the compositional correlations between RBPs and their target mRNAs in eCLIP data might be influenced or even created by cellular complexities such as RNA abundance, subcellular localization or protein–protein interactions. For reference, only slightly over half of the eCLIP CLIPper binding sites contain a known sequence motif for the RBP under investigation [67]. To address this, we turned to the High-Throughput RNA SELEX (HTR-SELEX) dataset from Jolma *et al*. [58], which is the only comparable large-scale *in vitro* study that predominately investigates RBPs in their native, full-length form. Unlike eCLIP, which is performed in living cells, HTR-SELEX captures RBP–RNA interactions under controlled biochemical conditions, thus eliminating numerous *in vivo* confounding variables.

**Figure 5.**
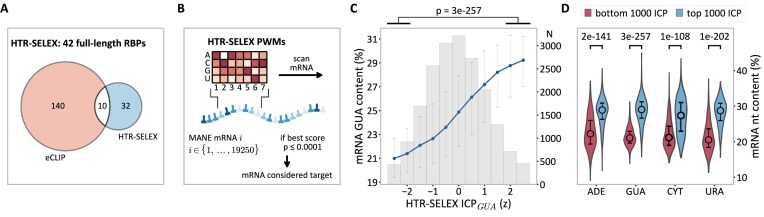
*In vitro* validation of compositional trends. (**A**) Overlap between RBPs studied via eCLIP (*N* = 150) and those studied by HTR-SELEX at full length (*N* = 42). (**B**) Schematic representation of the scanning procedure applied to the human transcriptome (19 250 MANE select transcripts) using PWMs; shown here for illustrative purposes is a heptamer PWM. (**C**) Histogram of HTR-SELEX-derived mRNA ICP_GUA_ (*N* = 19 158, bin-width = 0.5, right-hand *y*-axis) and the corresponding bin-median mRNA GUA-content (blue dots, left-hand *y*-axis). Error bars represent the associated interquartile ranges. The *P*-value above the panel refers to the statistical significance of the difference in the GUA-content between the sets of the top and the bottom 1000 mRNAs, as ranked according to ICP_GUA_ (Mann–Whitney *U* test). The corresponding *x*-axis ranges are indicated by the horizontal lines above. (**D**) Distributions of nucleotide contents (ADE, GUA, CYT, or URA) of the top and the bottom 1000 mRNAs, as ranked according to ICP for the respective nucleotide. The *P*-values above the violin-plot pairs refer to the statistical significance of the differences (Mann–Whitney *U* test).

Specifically, we analyzed 42 full-length RBPs from the HTR-SELEX dataset, only 10 of which overlap with the eCLIP set (Fig. 5A). For each RBP, we scanned the transcriptome (19 250 MANE transcripts) using both its primary and secondary target linear motifs as determined by HTR-SELEX (PWMs of 7 to 21 nucleotide widths), calling binding sites above a default *P* ≤ 0.0001 threshold (see Materials and methods). Any transcript meeting this criterion for at least one motif was considered a putative binder (Fig. 5B). While reproduction of individual eCLIP mRNA ICP values was poor (Pearson correlations between 0.02 and 0.08 between eCLIP and HTR-SELEX), binning the mRNAs in 1% nucleotide content intervals lead to correlations in ICP values of 0.87, 0.90, 0.96, and 0.97 for ADE, GUA, CYT, and URA, respectively. This indicates substantial noise at the individual mRNA level but confirms the robustness of the overarching composition-dependent binding behavior.

Strikingly, the same compositional biases seen in eCLIP data, but with an even higher statistical significance, were seen for HTR-SELEX data. For example, the top 1000 transcripts by ICP_GUA_ display a median GUA content of 29.1%, whereas the bottom 1000 are significantly lower at 21.1% (*P* ≤ 10^−258^, Mann–Whitney *U* test; Fig. 5C). Comparable separation holds for all four nucleotides, with *P*-values spanning 10^−107^ to 10^−258^ (Fig. 5D and Supplementary Fig. S15). These results suggest that the above patterns indeed reflect RNA–protein biophysics and are relatively independent of the cellular environment and constraints related to gene expression or localization.

### Compositional biases have functional implications: nuclear speckles as a case study

**Figure 6.**
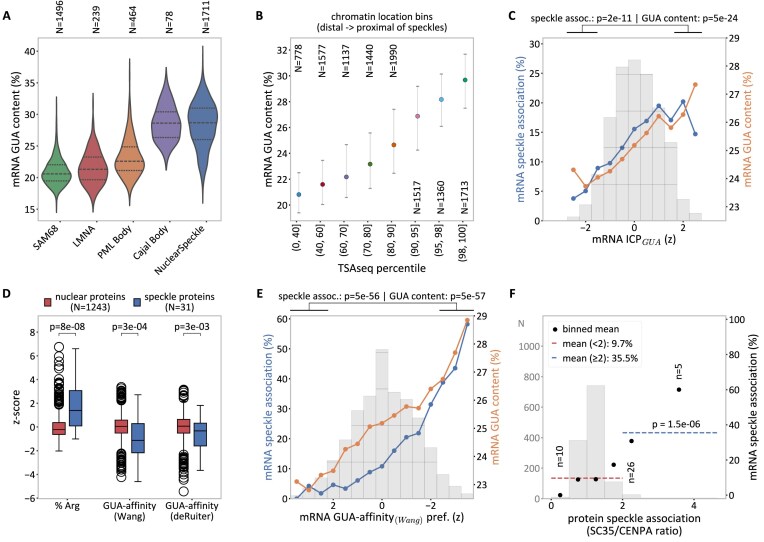
Compositional gradients toward nuclear speckles. (**A**) Distributions of mRNA GUA content in different nuclear domains defined by the APEX2 dataset [42], with indicated median and IQR. (**B**) Median and IQR GUA content for mRNAs binned by their genetic locus’ TSAseq [40] percentile: higher percentiles indicate more speckle-proximal chromatin. (**C**) Histogram of mRNA ICP_GUA_ distribution (bin width = 0.5). The gray blocks indicate the number of mRNAs in each bin (multiples of 500). The blue line represents bin-mean speckle association (%) and the orange line represents bin-median mRNA GUA-content (%). *P*-values refer to the difference in speckle association and GUA content between the bottom and top 500 mRNAs (Mann–Whitney *U* test), with corresponding *x*-axis ranges indicated by horizontal lines. (**D**) Detected proteins were grouped by TSA-MS SC35/SENPA ratio [65] using a cutoff of 2.0 (speckle: blue, other nuclear: red) and box plots indicate group median and IQR arginine composition (%) and peptide-mean amino acid GUA-affinities derived by bead-based fluorescence assays (Wang [63]) and molecular simulations (de Ruiter [64]). (**E**) Histogram of mRNA GUA-affinity preference (*z*) distribution, representing the statistical propensity of mRNAs to interact with RBPs based on their GUA-affinity-scaled (Wang [63]) amino acid compositions. The gray blocks indicate the number of mRNAs in each bin (multiples of 500). The blue line represents bin-mean speckle association (%) and the orange line represents bin-median mRNA GUA-content (%). *P*-values refer to the difference in speckle association and GUA content between the bottom and top 500 mRNAs (Mann–Whitney *U* test), with the corresponding *x*-axis ranges indicated by horizontal lines. (**F**) Histogram of speckle association values (TSA-MS SC35/SENPA ratio [65]) for 1274 detectable proteins with frequency (left-hand *y*-axis) and their autogenous mRNA speckle association [42] (means of bins; right-hand *y*-axis) indicated. Proteins with association values exceeding 2.5 (*N* = 5) were represented as a single bin, and were mapped to the corresponding mean *x* position. The red and blue horizontal lines capture the mean mRNA speckle association above (*N* = 31) versus below (*N* = 1243) a 2.0 cutoff in their autogenous protein’s SC35/SENPA ratio. The *P*-value refers to the corresponding Mann–Whitney *U* test between the two groups.

It was recently suggested that the human genome is organized along a speckle-to-lamina axis with a gradient in sequence composition [39]. This raises the question of how the compositional binding trends reported herein relate to the spatial organization of the nucleus. Notably, prior findings indicate that mRNAs with short introns enriched in GUA and CYT are preferentially localized to nuclear speckles [42, 43]. To explore this further, we analyzed the APEX2 dataset [42], representing mRNAs by their dominant, spliced (MANE) sequences, and found marked differences in their exonic GUA content across nuclear domains (Fig. 6A). Of particular importance is the increased GUA content (29%) of speckle mRNAs as compared to nuclear lamina-associated mRNAs (21%). In fact, GUA content is a more powerful predictor of mRNA speckle association than overall G/C or CYT content (Supplementary Fig. S16A–C). Furthermore, when we binned the human MANE transcriptome by the physical proximity of their genomic loci to nuclear speckles, given as percentiles in TSA-seq data [40], we observed a gradual increase in mRNA GUA content from 21% to 30% along the lamina-to-speckle axis (Fig. 6B).

We hypothesized that this nuclear compositional gradient is coupled to the compositional specificities within the mRNA–protein interaction network. Analyzing whether ICP predicts speckle association revealed a strong relationship, foremost for ICP_GUA_ (Fig. 6C): the more an mRNA prefers to interact with proteins encoded by high-GUA transcripts, the higher its probability of being speckle-associated. This is evidenced by a gradual increase in the speckle-associated mRNA population from <5% to ∼20% across ICP_GUA_ values ranging from −2.5 to 2.5.

This relationship likely stems from the specific composition of speckle proteins, which frequently feature arginine-rich, low-complexity regions [44]. Arginine codons are heavily GUA-biased (mean codon content: 1.33 GUA, 0.83 CYT, 0.67 ADE, and 0.17 URA), and substantial physicochemical evidence including aptamer studies [52], nucleobase/amino-acid statistical potentials [49], bead-based fluorescence affinity assays [63], and molecular dynamics simulations [64] demonstrates that arginine has a strong intrinsic affinity for guanine. Specifically, the 31 high-confidence speckle RBPs, defined by a cutoff of 2.0 in SC35/SENPA ratio TSA-MS [65], are significantly enriched in arginine content, exhibiting a mean of 10.6% (median 9.9%), compared to a mean of 6.0% (median 5.5%) for other detected nuclear proteins (*P *= 8 × 10⁻⁸, Fig. 6D). Importantly, this shifts the intrinsic physicochemical properties of speckle proteins as a whole. By scaling amino acid compositions to calculate a mean GUA affinity per protein, using interaction scales for all 20 amino acids derived from bead-based fluorescence assays in kcal/mol (Wang [63]) or molecular dynamics simulations in kJ/mol (de Ruiter [64]), we observe that speckle proteins possess significantly stronger overall GUA-affinities (lower binding energies) than the remaining nuclear proteome (*P *= 3 × 10^-4^ and *P *= 3 × 10⁻³, respectively; Fig. 6D).

To determine if this density of GUA-preferring amino acids explains the recruitment of G/C-rich mRNAs to speckles, we evaluated several plausible covariates. We first set a baseline by assessing whether an mRNA’s direct binding to speckle-associated RBPs predicts its speckle association, using APEX2 proximity labeling data [42] as a ground truth. Among the 150 profiled eCLIP RBPs, seven are established high-confidence speckle-associated proteins [65], including SRSF7, one of the marker proteins used for APEX2 proximity labeling. Reinforcing the proposed mechanism, these seven exhibit an increased arginine content as compared to the remaining 143 RBPs (mean arginine content of 12.6% versus 6.6%). For each mRNA, we computed a *z*-score representing its binding preference toward these seven proteins, jointly or specifically toward SRSF7, relative to randomized backgrounds (see Materials and methods section). While direct binding to SRSF7 alone is a surprisingly poor predictor of mRNA speckle association (*P *= 0.05, Supplementary Fig. S16D), combining the binding data for all seven eCLIP high-confidence speckle RBPs drastically improves predictive power. As *z*-scores for this joint measure rise from −2 to 2.5, speckle association probability rises from ∼5% to ∼48% (Supplementary Fig. S16E), and comparing the bottom and top 500 mRNAs by this measure reveals significant increases in both speckle association (*P *= 7 × 10⁻³⁵) and GUA content (*P *= 8 × 10^-9^). Intriguingly, an mRNA’s preference for arginine, quantified by the normalized mean arginine content of its bound eCLIP proteins, is an even better predictor of speckle association (*P *= 3 × 10^-39^; Supplementary Fig. S16F), indicating successful extrapolation to arginine-rich speckle proteins. Building on this, we evaluated each mRNA’s statistical preference to interact with RBPs according to their bulk GUA affinities. As expected, the more negative an mRNA’s set of bound RBPs is in its average GUA affinity, the higher the mRNA’s own GUA content (Fig. 6E and Supplementary Fig. S16G). Crucially, this GUA-affinity preference emerged as an extremely robust predictor of speckle association (Fig. 6E and Supplementary Fig. S16G), far outperforming raw intrinsic GUA content, transcript length, or expression (Supplementary Fig. S16). The top 500 mRNAs sorted by GUA-affinity preference showed significantly higher speckle association than the bottom 500 (*P *= 5 × 10⁻⁵⁶; Fig. 6E), yielding a 20.2-fold enrichment compared to only an 8.7-fold enrichment when sorting by intrinsic GUA content. At the absolute extremes, comparing the top 100 to the bottom 100 mRNAs reveals a 28.5-fold enrichment for GUA-affinity preference versus just 8.0-fold for intrinsic GUA content.

Interestingly, when predicting the physical proximity of the genomic locus to nuclear speckles (TSA-seq [40]), the predictive trend inverts. Intrinsic mRNA GUA-content strongly predicts chromatin closeness (*P *= 10⁻¹³³, Supplementary Fig. S16B), whereas GUA-affinity preference shows a weaker association (*P *= 2 × 10⁻¹² for Wang; *P *= 2 × 10^-9^ for de Ruiter; Supplementary Fig. S16G). This contrast indicates that mRNA speckle enrichment is not strictly a passive consequence of transcription at a speckle-proximal locus. While raw sequence composition correlates heavily with spatial proximity, actual transcript recruitment is better approximated by GUA-affinity preference. Because intrinsic GUA content cannot account for RNA secondary structure or competing protein interactions, an mRNA’s *in vivo* binding profile might serve as a more accurate measure of its functionally accessible GUA regions.

Finally, connecting to our previous finding that mRNAs and proteins of the same gene tend to bind each other [56], we plot the speckle enrichment of nuclear proteins (*N* = 1274 detected near centromere and speckles), as determined by TSA-MS [65], against speckle enrichment [42] of their mRNA counterparts (Fig. 6F). If binding and colocalization were in a causal relationship, one would expect to see a gradual increase in mRNA speckle association with increasing protein speckle enrichments, which we indeed observe (Fig. 6F). The top 30 proteins by nuclear speckle enrichment (SC35/SENPA ratio) were reported to consist of 96% validated speckle proteins [65], which equates approximately to a cutoff of 2.0 in SC35/SENPA ratio. The autogenous mRNAs of proteins that meet this cutoff are significantly more speckle-associated (35.5%) than the rest (9.7%), with a *P*-value of 1.5 × 10^−6^ (Mann–Whitney U test) (Fig. 6F).

## Discussion

**Figure 7.**
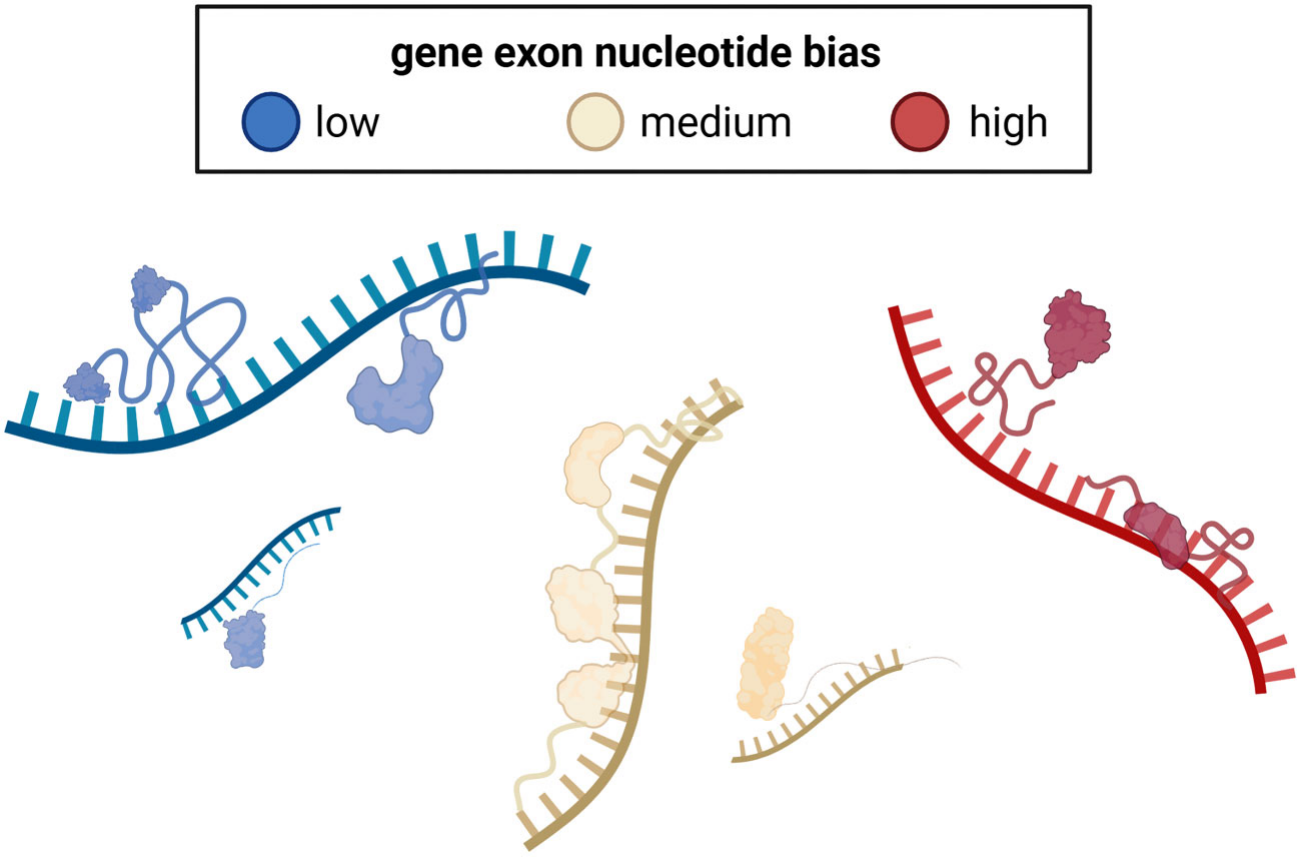
Nucleotide composition as a determinant of the large-scale structure of the cellular mRNA–protein interaction network. An mRNA’s composition mirrors how its bound proteome is on average encoded. Created in BioRender. Kapral, T. (2026) https://BioRender.com/b04e377.

Our study uncovers a simple organizing principle behind the large-scale structure of the human RNA–protein interaction network: the average nucleotide composition of an mRNA reflects the average composition of the transcripts that encode its bound RBPs. Equivalently put, compositional biases in the gene exon content of mRNAs tend to mirror those of their bound RBPs (Fig. 7). To ensure that the comparisons between different RBPs are not confounded by protocol-specific artefacts, we confined our in-cell analysis to the ENCODE eCLIP dataset, in which all 150 RBPs were profiled in HepG2 and K562 cells using an identical experimental workflow. This has ensured that any differences we detect originate from the properties of the RBPs themselves rather than any variability in experimental protocol. Furthermore, covariate-controlled analyses (Supplementary Fig. S6) demonstrate that these genome-wide trends are not driven by shared expression contexts, length biases, or binding promiscuity. In fact, the magnitude of the compositional shift is preserved or even magnified when strictly controlling for these variables. Crucially, the same compositional signal is recapitulated *in vitro* with HTR-SELEX data, indicating that it stems from the intrinsic properties of RNA-protein recognition rather than biases linked to cellular co-localization or expression.

The present findings place compositional matching alongside classical motif-based specificity as a second global axis of interactome organization. This distinction is firmly supported by our motif-stripped analysis (Supplementary Figs S8 and S9), which shows that the global correlations between mRNA nucleotide content and ICP remain highly significant even when all eCLIP-binding sites overlapping with linear sequence motifs are strictly removed. Indeed, compositional and motif-driven recognition could synergize in a dual-layer mechanism. Highly specific interactions that govern processes such as splice-site selection rely on folded RBDs, which engage short motifs. In parallel, intrinsically disordered regions (IDRs) and low-complexity segments like those rich in arginine and serine could provide numerous weak contact points whose cumulative strength directly contributes to the recognition and colocalization with the RNA partner [50].

The above findings are consistent with and can be rationalized by the idea of a stereochemical origin of the genetic code, which is rooted in the intrinsic, preferential interactions between amino acids and the nucleotides in their cognate codons [47, 51–53]. The extrapolation of this idea suggests that mRNAs preferentially interact with their own autogenous proteins [49–51, 54, 55], but also other proteins that are encoded by compositionally similar mRNAs [51], as demonstrated herein. The fact that the strongest auto-compositional matching arise from full target mRNA sequences is consistent with RBPs binding to both CDS and UTRs. It is less clear why full RBP transcripts show the strongest effects, since the explanation based on genetic code structure applies only to CDS. As UTRs encode information on transcript abundance, localization and functional grouping, these factors may also reflect bulk compositional trends linked to coding-related biases. Future research should clarify these relationships. Recent work has demonstrated a collective autoregulatory protein-RNA circuit that drives “interstasis” [38]. In this targeted homeostatic feedback loop, the accumulation of arginine-enriched proteins in condensates induces the selective sequestration of their own purine-rich multivalent transcripts to prevent dosage toxicity. Whereas interstasis acts as a specific, collective regulatory valve for highly dosage-sensitive, condensation-prone proteins, our transcriptome-wide analysis suggests that auto-compositional matching represents a broader, fundamental layer of interaction specificity, spanning the entire RNA–protein network and all four nucleotides. The present transcriptome-wide analysis gives support to this possibility and links the structure of the genetic code with the organization of the mRNA–protein interactome in today’s human cell. Finally, the present results make an important prediction that the amino-acid composition of RBPs may be reflected in the amino-acid composition of the proteins that their target mRNAs encode. Analysis of this potentially far-reaching prediction will be reported elsewhere.

Our analysis further connects sequence composition to nuclear geography, using nuclear speckles as a case study for how auto-compositional binding translates into spatial organization. While prior studies describe a general GUA/CYT gradient for both chromatin and RNA along the lamina-to-speckle axis [39, 42, 43], our dual analysis of locus proximity and transcript localization disentangles these phenomena. We propose that while intrinsic sequence features like GUA-content mark a gene’s spatial proximity to speckles, an mRNA’s statistical preference to bind RBPs with a high GUA-affinity provides the cooperative, physicochemical driving force for its direct recruitment into speckle condensates (Fig. 6C and E). This functional link is mirrored at the protein level, where autogenous mRNAs of speckle-enriched proteins are themselves disproportionately speckle-associated (Fig. 6F). Crucially, the high predictive power of an mRNA’s arginine-binding preference (Supplementary Fig. S16F), and ultimately its broader GUA-affinity preference (Fig. 6E), in assigning its speckle association aligns with extensive evidence that arginine preferentially interacts with guanine [49, 52, 63, 64], which predominates in its own codons. This should be viewed as an illustrative, not exclusive, example. Nuclear speckles are packed with RS-rich, mixed-charge domain proteins in which arginine is both highly abundant and conformationally accessible [44, 68], both of which factors support direct arginine/guanine recognition. Nevertheless, nucleobase/amino-acid affinity scales [47–49, 64, 69] reveal a general trend for many amino acids to preferentially interact with nucleotides in their own codons. This physical mechanism is highlighted by our analyses applying experimentally and computationally derived nucleobase-affinity scales (Fig. 6E and Supplementary Fig. S16G). An mRNA’s statistical preference to interact with RBPs, based specifically on their composition-defined GUA affinities, proved to be the most robust predictor of speckle association, outperforming raw transcript composition and more effectively capturing the cooperative dynamics of speckle recruitment. We, thus, interpret the role of arginine in the case of speckles to be a manifestation of a broader pattern: the combined effect of many parallel interactions between amino acids and the nucleotides that predominate in their codons is likely to underlie the global composition-matching trends reported here.

Several caveats merit notes. The present work surveys two cell lines and 150 RBPs—still only a subset of the full human repertoire. Although our primary analysis focused on mRNAs, we also conducted an exploratory evaluation of lncRNAs. Constrained by sparser detection and much lower interaction frequencies in the current dataset, this secondary analysis provided highly significant trends solely for cytosine, although the other nucleotides qualitatively point in the same direction. Given their statistical limitations, these findings remain preliminary, yet they provide an encouraging indication that the auto-compositional binding principle may extend beyond protein-coding transcripts. Moreover, compositional matching explains much, but not all, of the interaction network and necessarily coexists with motif- and structure-based recognition. Indeed, the cases where RBD-mediated specificity overrides the broader compositional biases (Fig. 4) likely arise due to the functional requirements of some RBPs to bind specific RNA motifs or secondary structures, regardless of nucleotide composition. Even so, the convergence of *in cell* eCLIP data, *in vitro* SELEX affinities and long-standing observations concerning the intrinsic nucleotide/amino-acid preferences establishes nucleotide composition and the auto-compositional binding principle as a *bona fide* axis of specificity. Incorporating this simple organizational principle should sharpen quantitative models of post-transcriptional regulation and help explain how linear sequence biases are transformed into the higher-order behavior of RNAs and proteins in three-dimensional cellular space.

## Supplementary Material

gkag540_Supplemental_Files

## Data Availability

The eCLIP dataset analyzed in this study was accessed through POSTAR3 (https://www.postar3.com/) [23], originally generated by the ENCODE Consortium [4, 22, 57]. Canonical transcript annotations were retrieved from the MANE database (version 1.2) [60], available at https://ftp.ncbi.nlm.nih.gov/refseq/MANE/, supplemented by ENSEMBL BioMart annotations (https://www.ensembl.org/biomart/martview) [61] for transcripts not covered by MANE. Proximity labeling datasets utilized in this study include mRNA-domain association data [42], TSA-seq chromatin proximity data [40], and TSA-MS protein-level speckle enrichment data [65], which were downloaded from the supplementary materials of the respective publications. HTR-SELEX data for 42 full-length RBPs were downloaded from supplementary tables provided by Jolma *et al*. (2020) [58]. Gene expression data were retrieved from the Human Protein Atlas (https://www.proteinatlas.org). Non-coding transcript coordinates were retrieved from GENCODE v49 (https://www.gencodegenes.org). Seven processed data files accompany this paper as Supplementary Data S1 contains nucleotide compositions together with eCLIP-derived ICP values for the 11 669 MANE transcripts that interact with at least one eCLIP RBP. Supplementary Data S2 contains nucleotide compositions together with HTR-SELEX-derived Interactome Codedness Preference values for the 19 158 MANE transcripts that interact with at least one HTR-SELEX RBP. Supplementary Data S3 lists nucleotide compositions for the MANE transcripts that encode the 150 eCLIP proteins. Supplementary Data S4 lists nucleotide compositions for the MANE transcripts that encode the 42 HTR-SELEX proteins. Supplementary Data S5 is a 150 × 11 669 binary matrix that marks interactions between the 150 eCLIP RBPs and their MANE targets. Data S6 is a 42 × 19 158 binary matrix that marks inferred interactions between the 42 HTR-SELEX RBPs and all MANE targets. Supplementary Data S7 is a 150 × 5 table of negative log₁₀ *P*-value shifts recorded after individually omitting each eCLIP RBP.
