# Supplementary material for: RNA-binding protein transcripts reflect composition of target mRNAs
Source: Nucleic Acids Res. 2026 Jun 8;54(11):gkag540. doi: 10.1093/nar/gkag540 (PMC13244147; doi:10.1093/nar/gkag540)
Supplement: gkag540_Supplemental_Files [file gkag540_supplemental_files.zip › Supplementary_Materials.pdf]

## SUPPLEMENTARY DATA

**Data S1:**

File contains nucleotide compositions (GUA, ADE, CYT, URA), eCLIP based Interactome Codedness Preference (ICP) values ($ICP_{GUA}$, $ICP_{ADE}$, $ICP_{CYT}$, $ICP_{URA}$) and other RBP feature preferences for the 11,669 MANE transcripts that interact with at least one RBP in this dataset.

**Data S2:**

File contains nucleotide compositions (GUA, ADE, CYT, URA) and HTR-SELEX based Interactome Codedness Preference (ICP) values ($ICP_{GUA}$, $ICP_{ADE}$, $ICP_{CYT}$, $ICP_{URA}$) for the 19,158 MANE transcripts that interact with at least one RBP in this dataset.

**Data S3:**

File includes nucleotide compositions (GUA, ADE, CYT, URA) of MANE transcripts that encode the 150 eCLIP proteins alongside other selected protein features.

**Data S4:**

File includes nucleotide compositions (GUA, ADE, CYT, URA) of MANE transcripts that encode the 42 HTR-SELEX proteins.

**Data S5:**

A binary matrix (11,669 x 150) indicating interactions between the 150 eCLIP RBPs and the 11,669 MANE transcripts. Rows represent transcripts, columns represent RBPs, while matrix values are 1 (indicating interaction) or 0 (indicating no interaction).

**Data S6:**

A binary matrix (19,158 x 42) indicating motif-derived interactions between the 42 HTR-SELEX RBPs and the 19,158 MANE transcripts. Rows represent transcripts, columns represent RBPs, while matrix values are 1 (indicating interaction) or 0 (indicating no interaction).

**Data S7:**

A 150 × 5 matrix of negative $\log_{10}$ p-value shifts observed when each eCLIP RBP is individually excluded, as visualized in Fig. 4B, quantifying their impact on the p-values plotted in Fig. S3. For every RBP the mean impact per nucleotide (G, A, C, U) and also the mean impact on all 8 p-values is given. High absolute values indicate high compositional specificities of RBPs that are positively (if positive) or negatively (if negative) their own mRNAs' composition bias.

**Data S8:**

A Python script (ICP_calculation.py) that calculates the ICP values ($ICP_{GUA}$, $ICP_{ADE}$, $ICP_{CYT}$, $ICP_{URA}$) for a given set of transcripts. The script processes an RBP nucleotide composition table (e.g., Data S3 or S4) and a binary RNA-protein interaction matrix (e.g., Data S5 or S6). It computes z-scores by comparing the mean nucleotide composition of actual bound RBPs against a simulated null distribution, outputting an N mRNA x 4 ICP matrix analogous to Data S1 and S2. It optionally also calculates mRNA binding preferences to other RBP features, like arginine content.

## SUPPLEMENTARY FIGURES

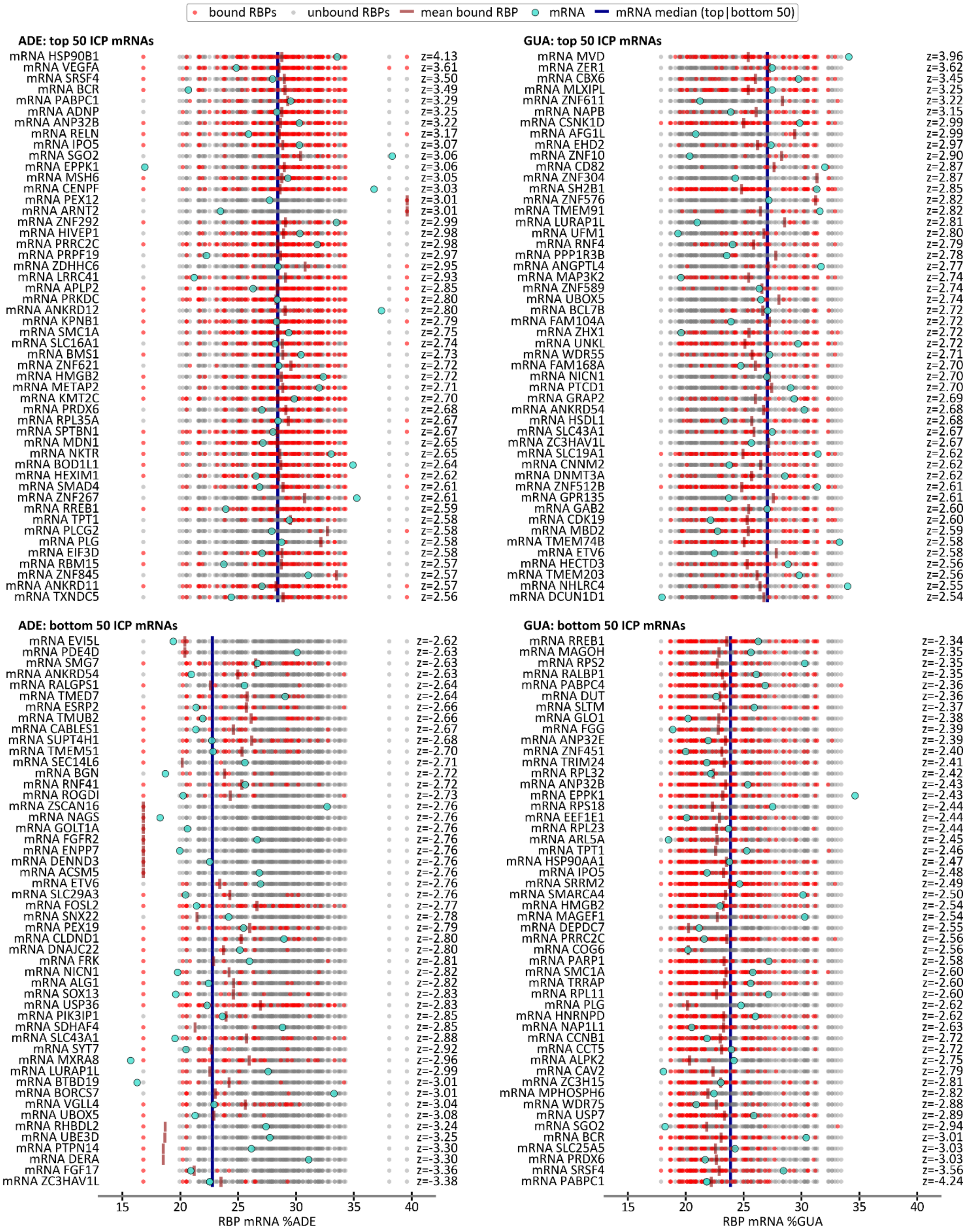


**fig. S1. Compositional biases in mRNA interactomes (ADE, GUA).** Encoding mRNA nucleotide contents (red dots) of the RBP interactomes of the top and the bottom 50 target mRNAs by ICP

value, for ADE and GUA. For a given target mRNA, the average nucleotide content of its interacting RBP mRNAs is depicted with red bars, while its own nucleotide content is given as a cyan dot. The average nucleotide content of the two respective target mRNA groups for each nucleotide type is indicated as blue vertical lines.

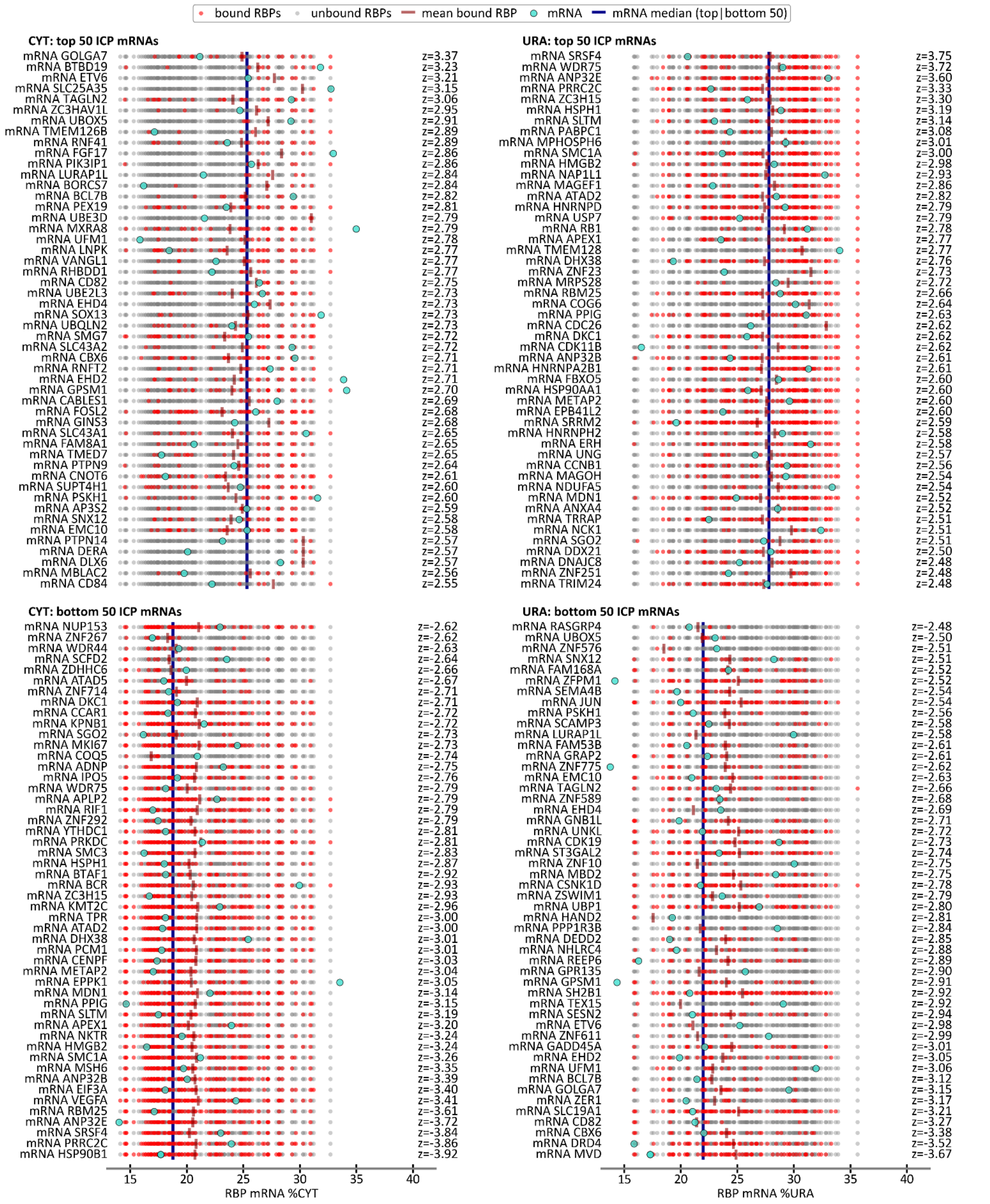


**fig. S2. Compositional biases in mRNA interactomes (CYT, URA).** Encoding mRNA nucleotide contents (red dots) of the RBP interactomes of the top and the bottom 50 target mRNAs by ICP value, for CYT and URA. For a given target mRNA, the average nucleotide content of its interacting RBP mRNAs is depicted with red bars, while its own nucleotide content is given as a

cyan dot. The average nucleotide content of the two respective target mRNA groups for each nucleotide type is indicated as blue vertical lines.

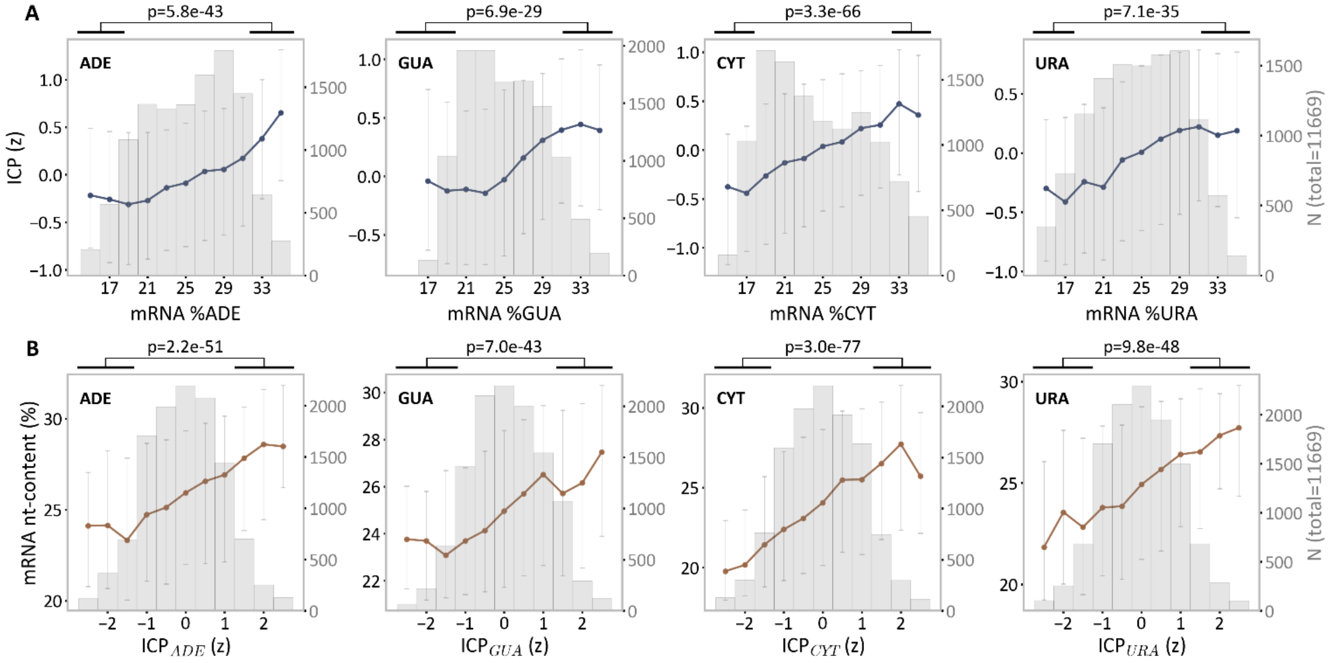


**fig. S3. mRNA nucleotide content is related to its ICP. (A)** *(left to right)* histograms of target mRNA nucleotide content (N=11,669; bin-width = 2%; right-hand y-axis) and the corresponding bin-median ICP (blue dots, left-hand y-axis), for all 4 standard RNA nucleotides. **(B)** histograms of mRNA ICP (N=11,669, bin-width = 0.5, right-hand y-axis) and the corresponding bin-median mRNA nucleotide content (orange dots, left-hand y-axis), for all 4 standard RNA nucleotides. Error bars represent the associated interquartile ranges. The p-values refer to the statistical significance of the difference in the y-axis variable between the sets of the top and the bottom 1000 mRNAs, as ranked according to the variable on the x-axis (Mann-Whitney U-test). The corresponding x-axis ranges are indicated by the horizontal lines above.

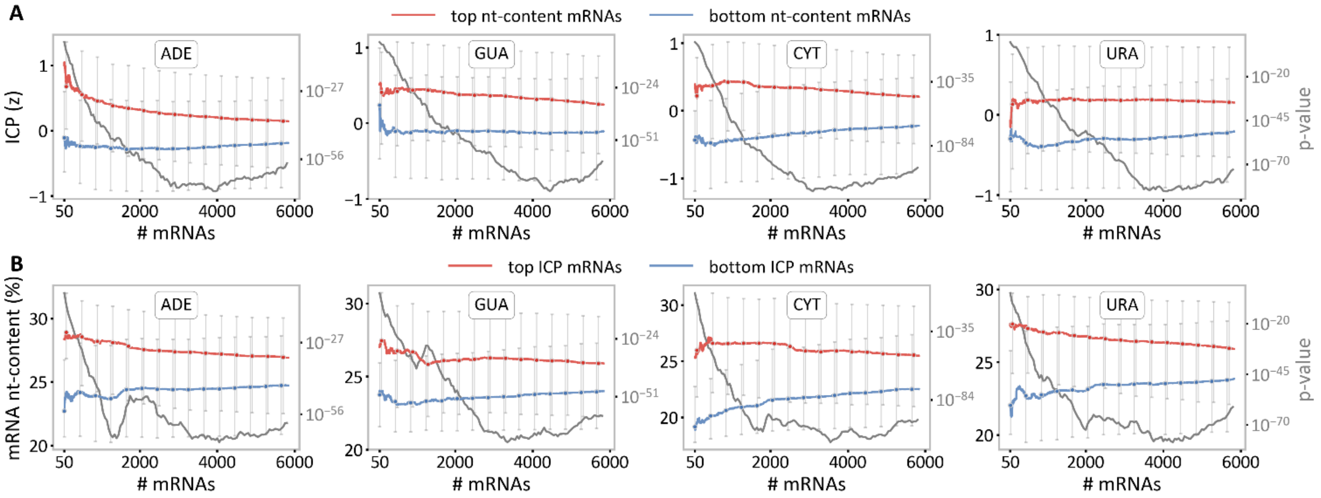


**fig. S4**: **Statistically significant separation between ICP and nucleotide-content extremes** **(A)** median ICP of the N mRNAs with the highest (red) and the lowest (blue) respective nucleotide content as a function of N, for all 4 RNA standard nucleotides. **(B)** median mRNA nucleotide content of the N mRNAs with the highest (red) and the lowest (blue) respective ICP as a function of N, for all 4 RNA standard nucleotides. In all panels, the error bars capture the interquartile ranges, while the associated p-values refer to the difference between the two sets at a given N as evaluated by Mann-Whitney U test.

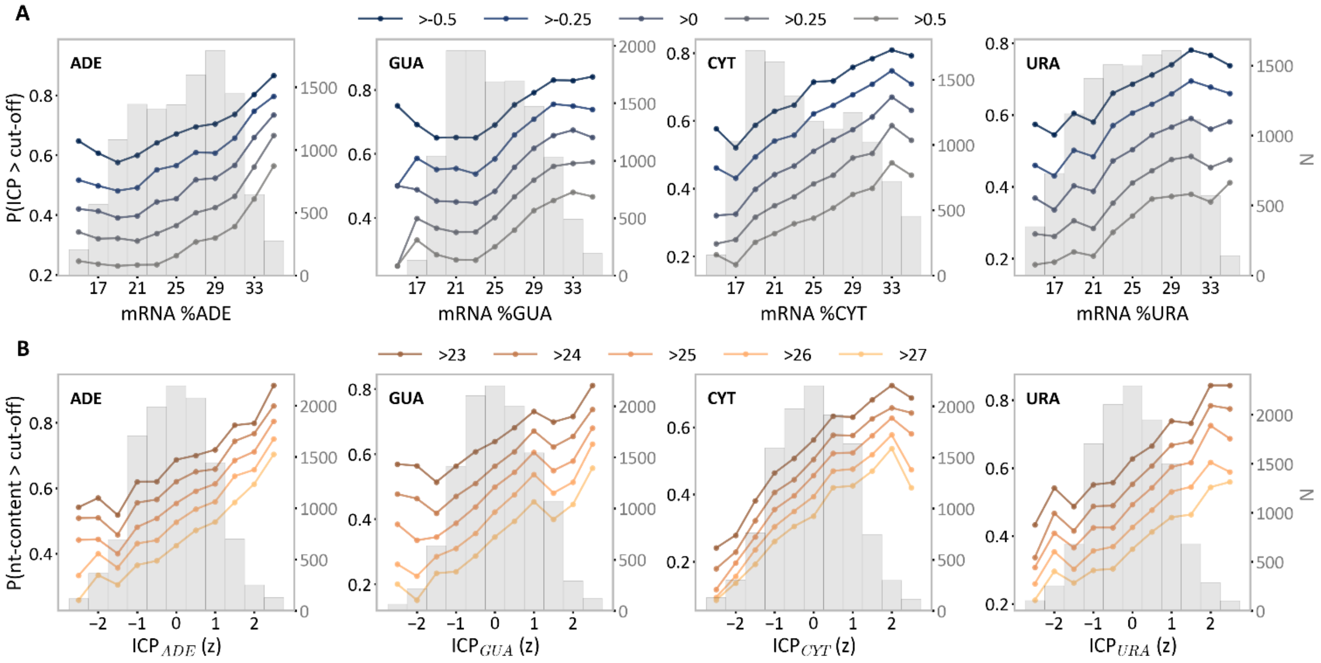


**fig. S5. Differentiated view of the relationship between mRNA nucleotide content and ICP.** **(A)** *(left to right)* histograms of mRNA nucleotide content (N=11,669, bin-widths = 2%, right-hand y-axis) with the corresponding fraction of mRNAs in a given bin that exceed a minimum ICP cutoff, as indicated above the panels (blue lines, left-hand y-axis), for ADE, GUA, CYT and URA. **(B)** histograms of mRNA ICP (N=11,669, bin-widths = 0.5, right-hand y-axis) and the corresponding fraction of mRNAs in a given bin that exceed a minimum nucleotide-content cutoff, as indicated above the panels (orange lines, left-hand y-axis), for ADE, GUA, CYT and URA.

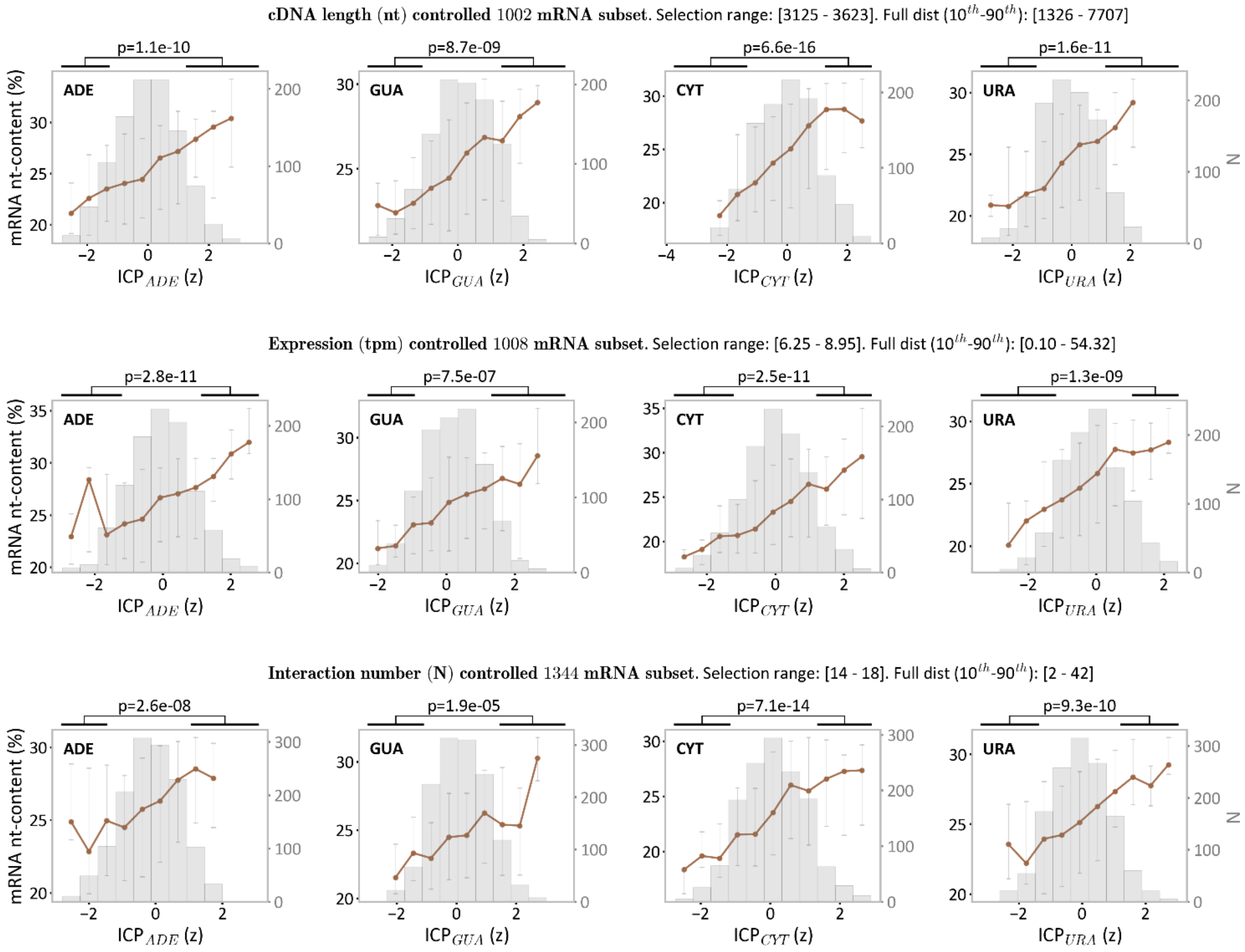


**fig. S6. Covariate-controlled analysis of ICP versus mRNA nucleotide content.** The relationship between mRNA ICP and nucleotide content was analyzed in three restricted subsets of transcripts to control for potential confounders. Each panel displays histograms of mRNA ICP (grey bars, right-hand y-axis) and the corresponding bin-median mRNA nucleotide content (brown dots, left-hand y-axis) for the four standard nucleotides. Rows correspond to subsets controlled for: (top) cDNA length (N=1,002; range: 3,125–3,623 nt); (middle) expression level (N=1,008; range: 6.25–8.95 tpm); and (bottom) number of bound RBPs (N=1,344; range: 14–18 interactions). The 10$^{th}$–90$^{th}$ percentile ranges of the full transcriptome distribution are provided in the row headers for comparison. Error bars represent interquartile ranges. P-values indicate the statistical significance of the difference in nucleotide content between the top and bottom 100 mRNAs ranked by ICP (Mann-Whitney U-test, 1-sided), with the corresponding x-axis ranges marked by horizontal brackets.

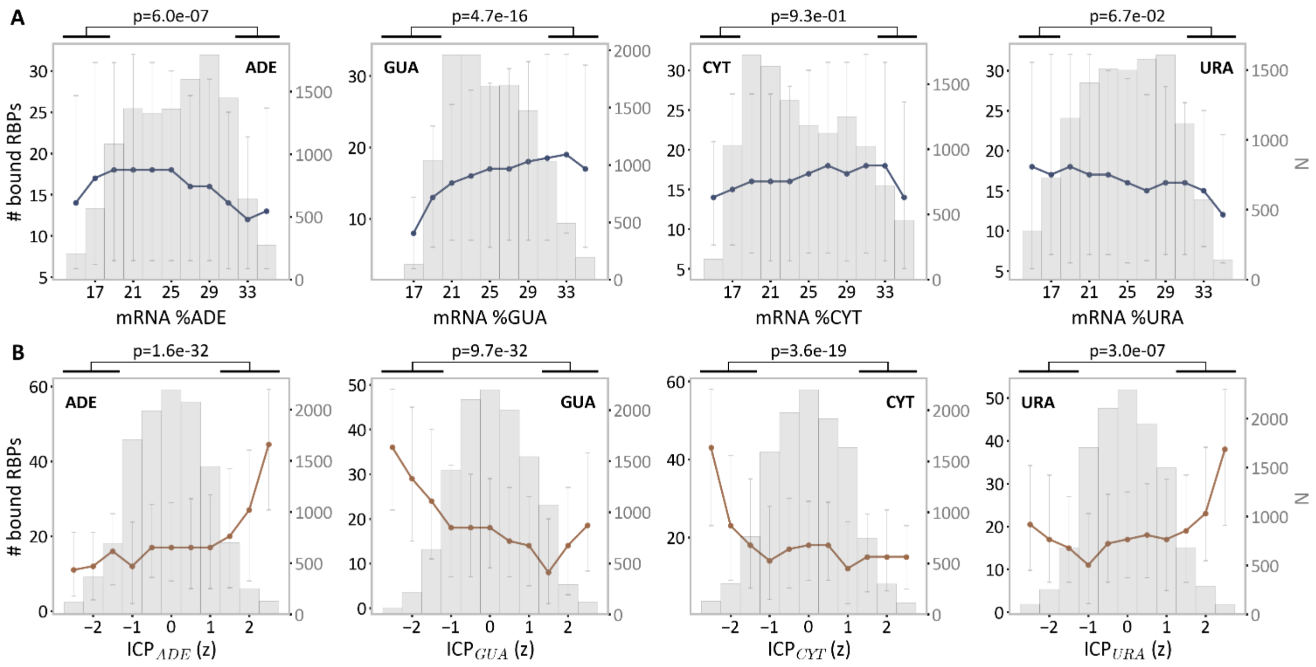


**fig. S7. Correlations between ICP and binding frequencies. (A)** histograms of mRNA nucleotide content (N=11,669, bin-width = 2%, right-hand y-axis) and the corresponding bin-median number of bound RBPs (blue dots, left-hand y-axis), for ADE, GUA, CYT and URA. **(B)** histograms of mRNA ICP (N=11,669, bin-width = 0.5, right-hand y-axis) and the corresponding bin-median number of bound RBPs (blue dots, left-hand y-axis), for ADE, GUA, CYT and URA. Error bars represent the associated interquartile ranges. The reported p-values were calculated for the top and the bottom 1000 mRNAs in each panel using the 2-sided Mann-Whitney U-test, whereby the corresponding x-axis ranges are indicated by the horizontal lines above.

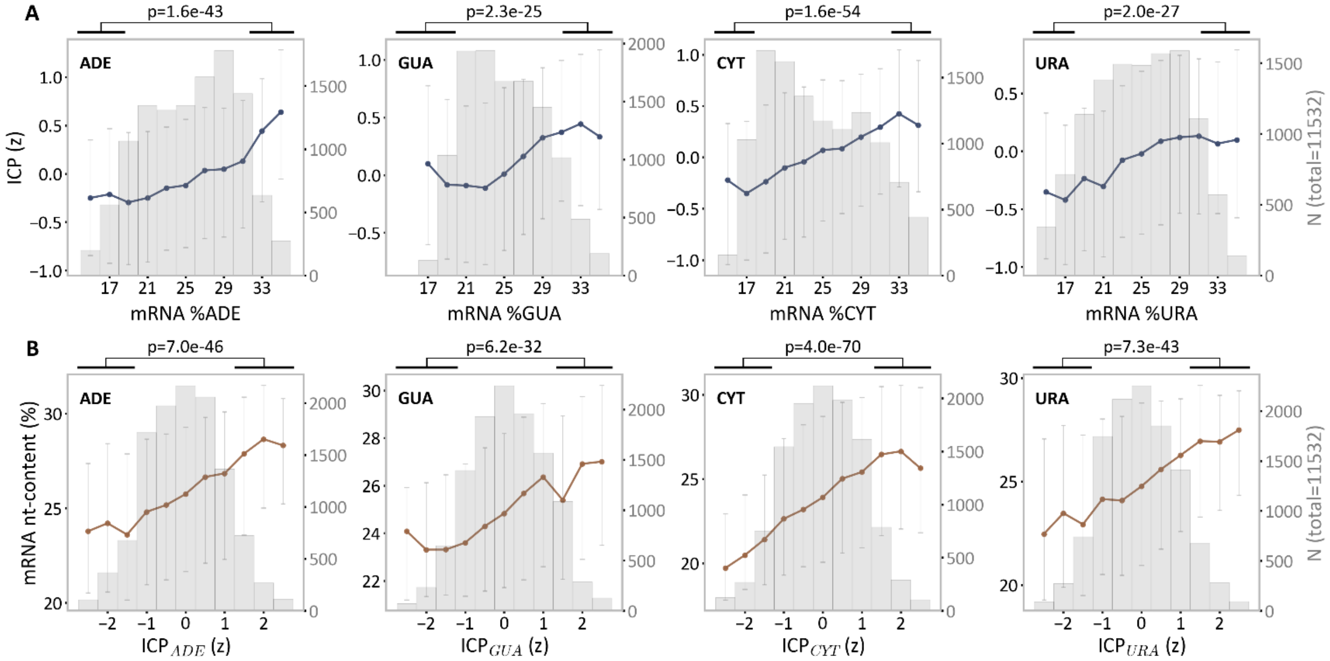


**fig. S8. Motif sequence-controlled analysis of ICP versus mRNA nucleotide content (default version).** This is a reproduction of results shown in fig. S3 after the removal of binding site residues that overlap with sequence motifs, utilizing both default motif discovery ($E < 0.05$) and default site allocation thresholds ($p < 0.0001$). **(A)** (left to right) histograms of target mRNA nucleotide content (N=11,532; bin-width = 2%; right-hand y-axis) and the corresponding bin-median ICP values (blue dots, left-hand y-axis), for all 4 standard RNA nucleotides. **(B)** histograms of mRNA ICP (N=11,532, bin-width = 0.5, right-hand y-axis) and the corresponding bin-median mRNA nucleotide content (orange dots, left-hand y-axis), for all 4 standard RNA nucleotides. Error bars represent the associated interquartile ranges. The p-values refer to the statistical significance of the difference in the y-axis variable between the sets of the top and the bottom 1000 mRNAs, as ranked according to the variable on the x-axis (Mann-Whitney U-test, 1-sided). The corresponding x-axis ranges are indicated by the horizontal lines above.

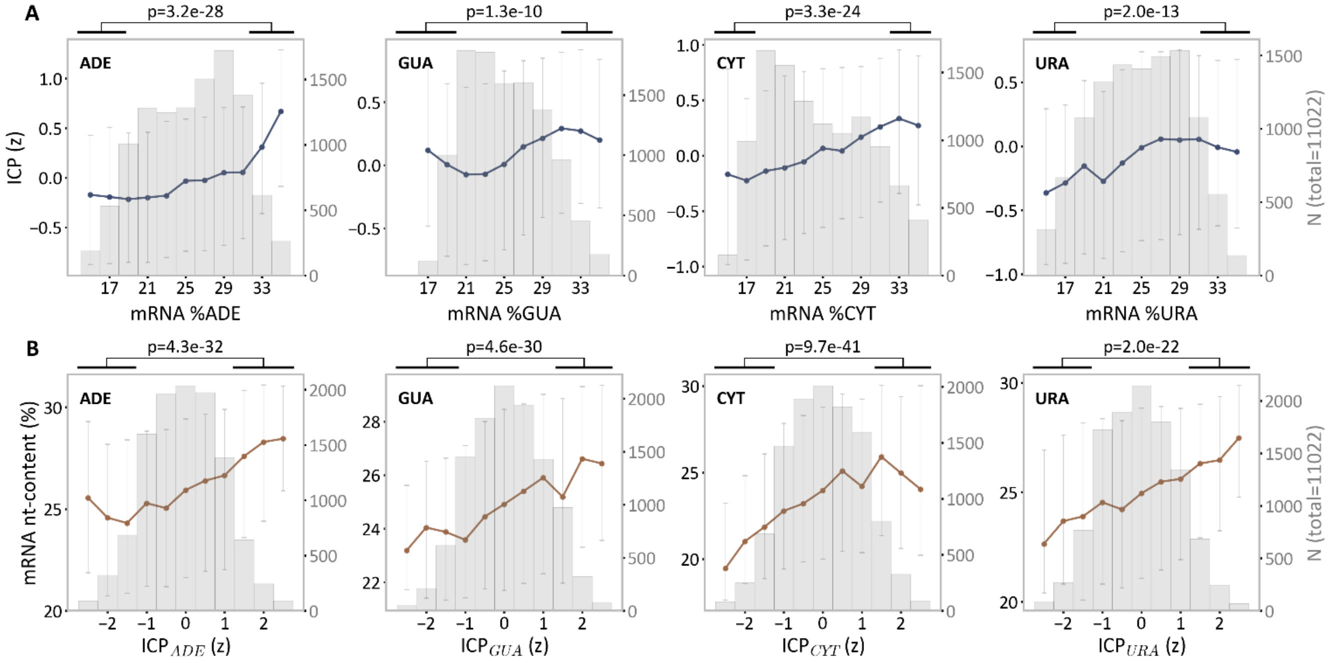


**fig. S9. Motif sequence-controlled analysis of ICP versus mRNA nucleotide content (strict version).** This is a reproduction of results shown in fig. S3 after the strict, broad removal of binding sites intersecting sequence motifs, utilizing both lenient motif discovery ($E < 0.2$) and lenient site allocation thresholds ($p < 0.001$). **(A)** (left to right) histograms of target mRNA nucleotide content (N=11,022; bin-width = 2%; right-hand y-axis) and the corresponding bin-median ICP (blue dots, left-hand y-axis), for all 4 standard RNA nucleotides. **(B)** histograms of mRNA ICP (N=11,022, bin-width = 0.5, right-hand y-axis) and the corresponding bin-median mRNA nucleotide content (orange dots, left-hand y-axis), for all 4 standard RNA nucleotides. Error bars represent the associated interquartile ranges. The p-values refer to the statistical significance of the difference in the y-axis variable between the sets of the top and the bottom 1000 mRNAs, as ranked according to the variable on the x-axis (Mann-Whitney U-test, 1-sided). The corresponding x-axis ranges are indicated by the horizontal lines above.

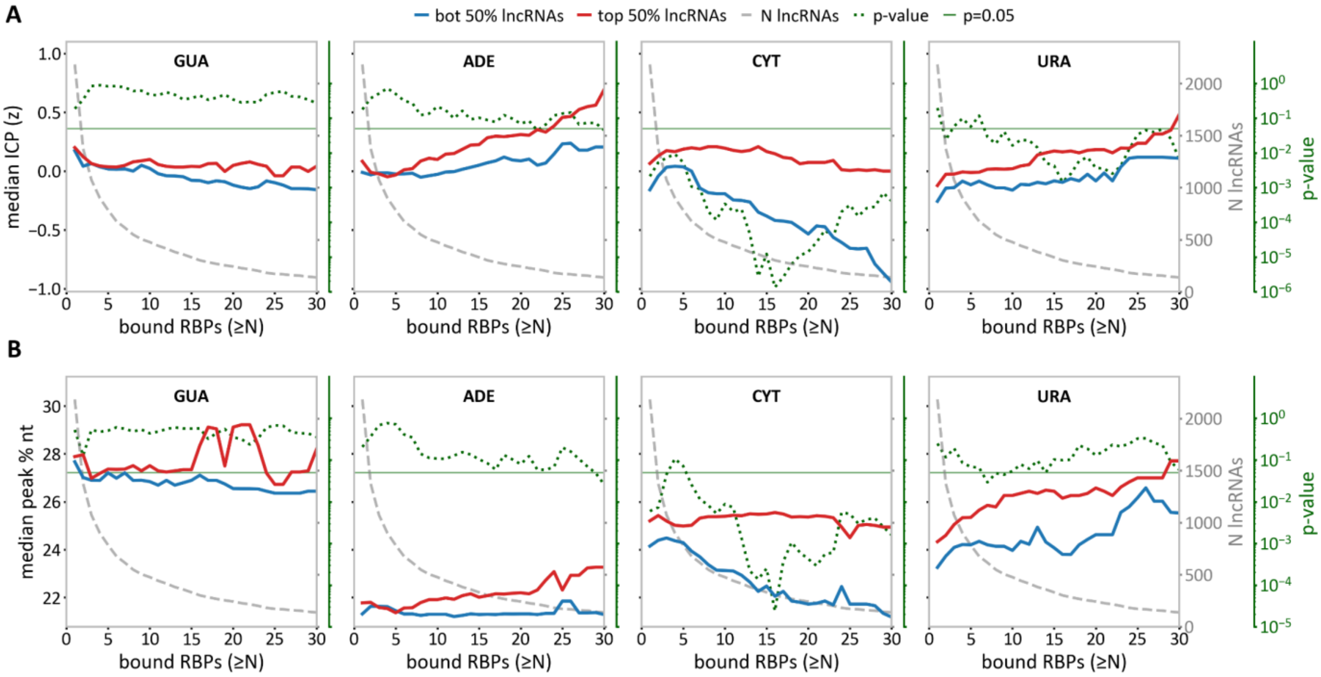


**fig. S10. Compositional matching in lncRNAs at different interaction counts**. The effect of applying a rolling minimum threshold of the number of bound RBPs (x-axis, 1 to 30) on the spread and statistical significance between bisected lncRNA cohorts. **(A)** Median ICP z-scores (left y-axis) for the top 50% (red line) and bottom 50% (blue line) of lncRNAs bisected by their intrinsic peak nucleotide content. **(B)** Median peak nucleotide content (left y-axis) for the top 50% (red line) and bottom 50% (blue line) of lncRNAs bisected by their ICP z-score. In both panels, the results are shown from left to right for GUA, ADE, CYT and URA, respectively. The secondary axes display the sample size (N lncRNAs, grey dashed line; first right-hand y-axis) and the corresponding one-sided Mann-Whitney U test p-value (green dotted line; second right-hand y-axis), which evaluates whether the respective value on the left-hand y-axis for the top cohort is stochastically greater than for the bottom cohort. The solid green line marks the p = 0.05 significance threshold.

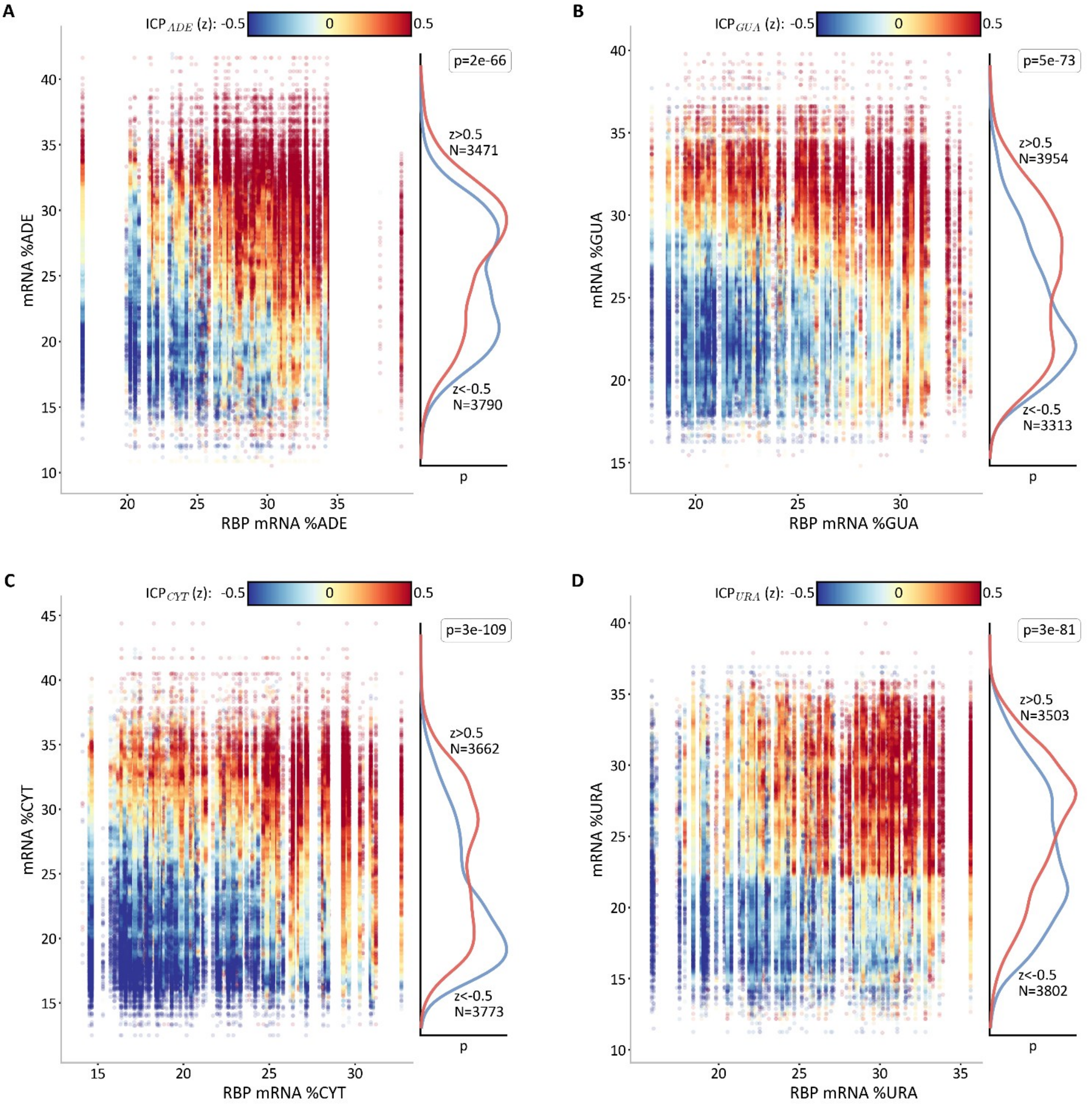


**fig. S11. Compositional gradients dominate the large-scale organization of the human RNA-protein interaction network.** eCLIP RNA-protein interactome (150 RBPs vs. 11,669 mRNAs), as represented by RBP mRNA and target mRNA nucleotide content for **(A)** ADE, **(B)** GUA, **(C)** CYT, and **(D)** URA. Dots corresponding to the RBPs interacting with a given mRNA (horizontal lines) are colored according to this mRNA's ICP (legend given above the graphs). Overlapping dots are shown in the same color, representing the mean of nearby dots within dx=0% and dy ≤ 0.5%. Distributions of nucleotide content of mRNAs with the respective ICP > 0.5 (red) and <-0.5 (blue), together with the associated p-values (Mann Whitney U, 2-sided), are given on the right. The number of mRNAs behind each distribution is given next to the curves.

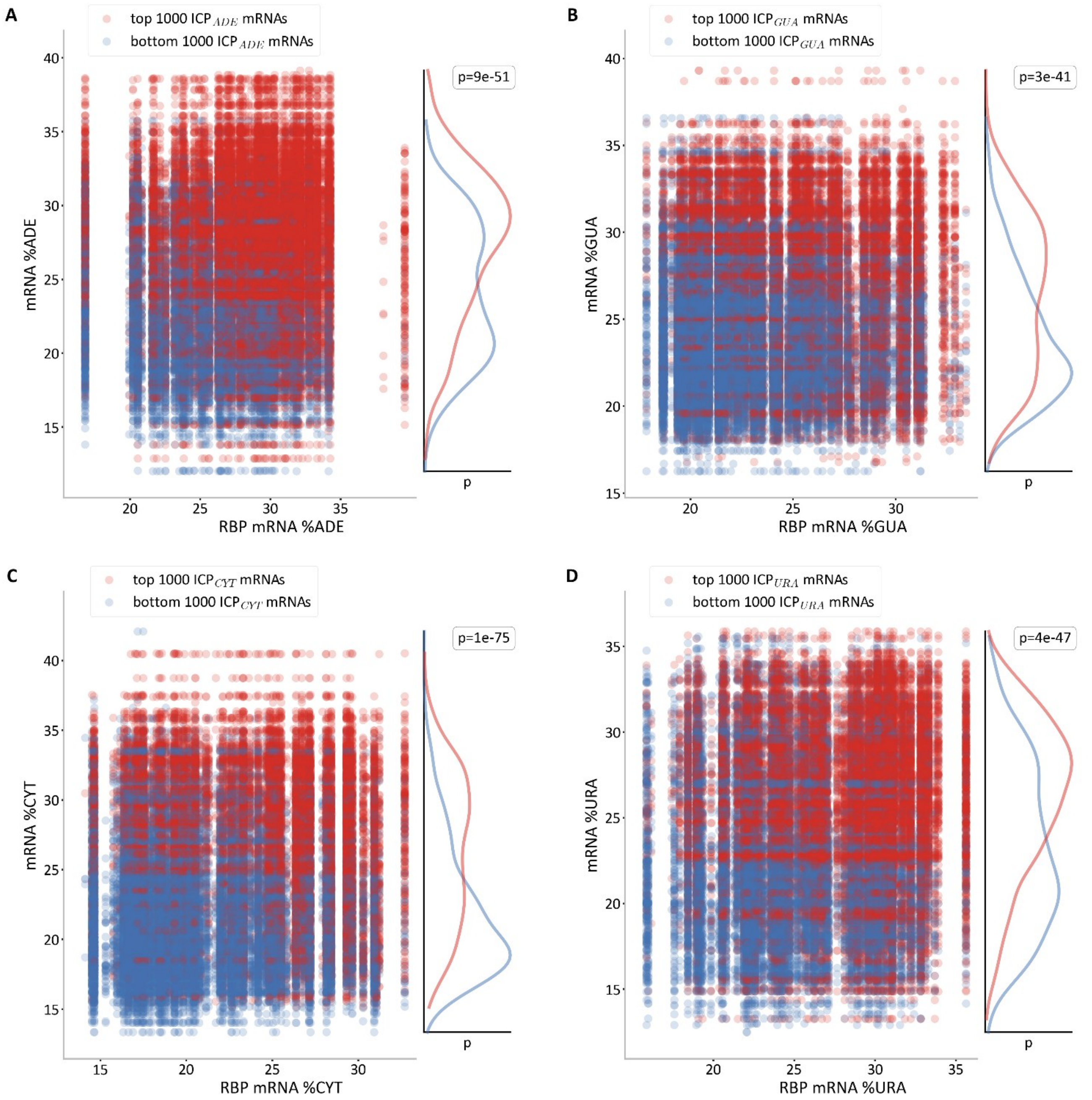


**fig. S12. Compositional gradients dominate the large-scale structure of the human RNA-protein interactome (filtered).** eCLIP RBP interactome for the top (red) and the bottom (blue) 1000 human mRNAs according to their ICP as represented by RBP mRNA and target mRNA nucleotide content for **(A)** ADE, **(B)** GUA, **(C)** CYT and **(D)** URA. Compositional densities of mRNAs in the two sets with the associated p-values (Mann-Whitney U-test, 2-sided) are given on the right of each panel. Dots are depicted with 80% transparency and z-ordered by absolute ICP.

**A**

**interactions with top 1000 ICP$_{ADE}$ mRNAs**

| | top 10 bound RBPs | top 10 enriched RBPs |
|---|---|---|
| 1 | UTP3 (669, +30%) | PPIL4 (54, +740%) |
| 2 | BCLAF1 (668, +60%) | SAFB2 (56, +415%) |
| 3 | DDX3X (636, +9%) | SAFB (50, +407%) |
| 4 | UCHL5 (635, +61%) | SSB (9, +377%) |
| 5 | PPIG (584, +35%) | FXR1 (186, +337%) |
| 6 | ZNF800 (554, +84%) | NPM1 (46, +319%) |
| 7 | PUM2 (531, +32%) | YWHAG (38, +303%) |
| 8 | LIN28B (530, +55%) | LARP7 (160, +250%) |
| 9 | AQR (518, +41%) | AGGF1 (208, +229%) |
| 10 | YBX3 (507, +22%) | ZC3H8 (15, +218%) |

**B**

**interactions with top 1000 ICP$_{GUA}$ mRNAs**

| | top 10 bound RBPs | top 10 enriched RBPs |
|---|---|---|
| 1 | UPF1 (620, +64%) | TAF15 (20, +332%) |
| 2 | UTP3 (563, +9%) | EWSR1 (63, +239%) |
| 3 | RBM15 (506, +29%) | HNRNPM (116, +235%) |
| 4 | DDX3X (455, -22%) | SFPQ (11, +213%) |
| 5 | PABPC4 (446, -10%) | BCCIP (5, +207%) |
| 6 | GRWD1 (424, -12%) | AKAP8L (117, +171%) |
| 7 | TBRG4 (377, +50%) | GTF2F1 (174, +152%) |
| 8 | BUD13 (363, -5%) | FKBP4 (37, +151%) |
| 9 | ZNF622 (360, +8%) | POLR2G (4, +146%) |
| 10 | DGCR8 (350, +42%) | HNRNPU (7, +140%) |

**C**

**interactions with top 1000 ICP$_{CYT}$ mRNAs**

| | top 10 bound RBPs | top 10 enriched RBPs |
|---|---|---|
| 1 | UPF1 (655, +74%) | PTBP1 (147, +151%) |
| 2 | PABPC4 (535, +9%) | BCCIP (4, +146%) |
| 3 | GRWD1 (489, +2%) | GTF2F1 (134, +94%) |
| 4 | YBX3 (480, +15%) | HNRNPUL1 (10, +91%) |
| 5 | DDX3X (469, -19%) | PCBP2 (374, +86%) |
| 6 | TBRG4 (413, +64%) | POLR2G (3, +84%) |
| 7 | RBM15 (391, +0%) | AKAP1 (329, +78%) |
| 8 | FAM120A (383, +50%) | UPF1 (655, +74%) |
| 9 | PCBP2 (374, +86%) | XPO5 (63, +71%) |
| 10 | UTP3 (371, -28%) | SF3B4 (212, +71%) |

**D**

**interactions with top 1000 ICP$_{URA}$ mRNAs**

| | top 10 bound RBPs | top 10 enriched RBPs |
|---|---|---|
| 1 | DDX3X (720, +24%) | PPIL4 (30, +367%) |
| 2 | BCLAF1 (681, +63%) | CPSF6 (188, +320%) |
| 3 | UCHL5 (611, +55%) | NPM1 (36, +228%) |
| 4 | GRWD1 (582, +21%) | FXR1 (127, +198%) |
| 5 | PUM2 (568, +42%) | YWHAG (26, +176%) |
| 6 | PPIG (536, +24%) | TRA2A (259, +172%) |
| 7 | LIN28B (533, +56%) | SRSF7 (151, +169%) |
| 8 | PABPC4 (529, +7%) | CPEB4 (182, +158%) |
| 9 | SUB1 (517, +105%) | TIAL1 (228, +138%) |
| 10 | RPS3 (507, +32%) | SBDS (8, +133%) |

**fig. S13. Relative RBP contribution to mRNA ICP.** Top 10 RBPs in terms of the absolute number of interactions (left) or enrichment (right) among the interactors of the top 1000 mRNAs as ranked by ICP for **(A)** GUA, **(B)** ADE, **(C)** CYT and **(D)** URA. The enrichment is calculated as the bound fraction among the top 1000 ICP mRNAs over the bound fraction among all mRNAs.

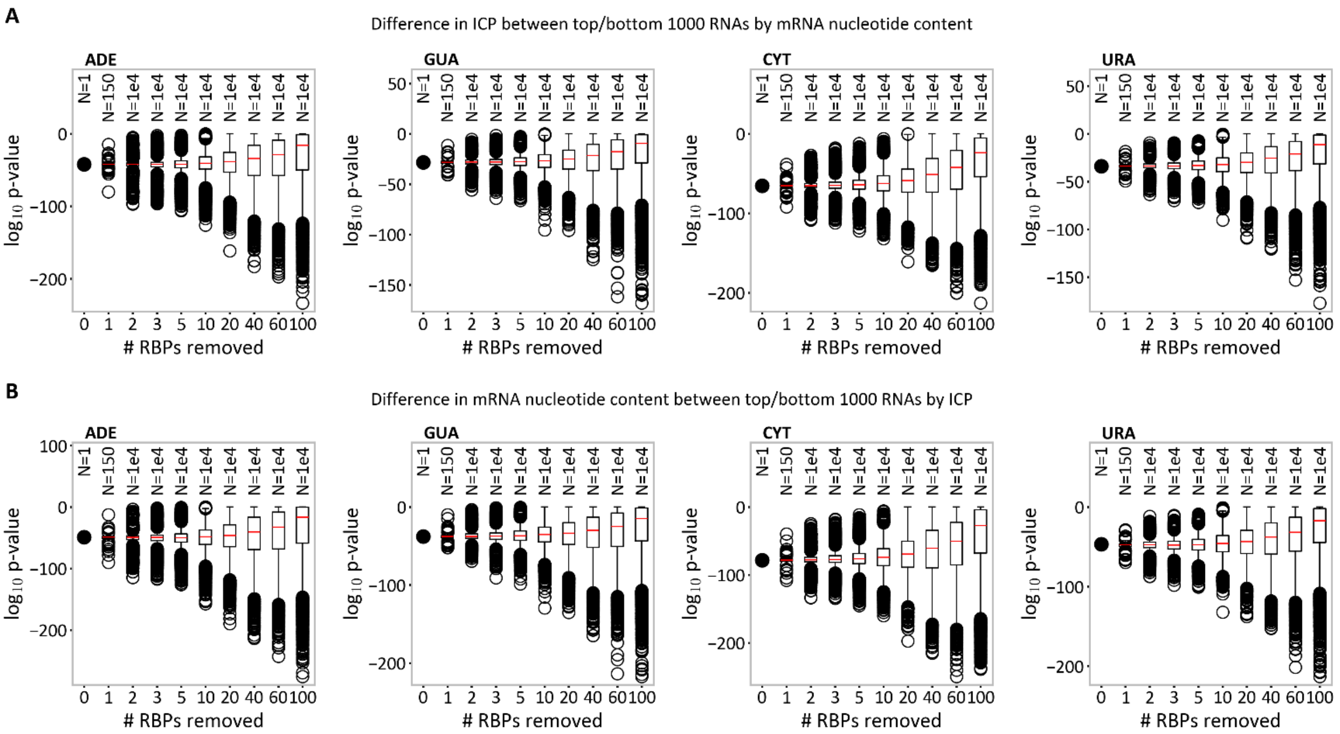


**fig. S14. Analysis of robustness to subsampling. (A)** log10 p-values (U-test, 1-sided) obtained after removing 0–100 out of 150 eCLIP RBPs and evaluating the statistical significance of the separation in ICP values between the top and the bottom 1000 mRNAs as ranked by the content of the respective nucleotide, for (left to right) GUA, ADE, CYT and URA. The x-axis indicates the number of RBPs removed. Red lines represent the median p-values of N randomization trails. **(B)** log10 p-values (U-test, 1-sided) obtained after removing 0–100 out of 150 eCLIP RBPs and evaluating the statistical significance of the separation in nucleotide content between the top and the bottom 1000 mRNAs as ranked by ICP values for the respective nucleotide, for (left to right) GUA, ADE, CYT and URA. The x-axis indicates the number of RBPs removed. Red lines represent the median p-values of N randomization trails.

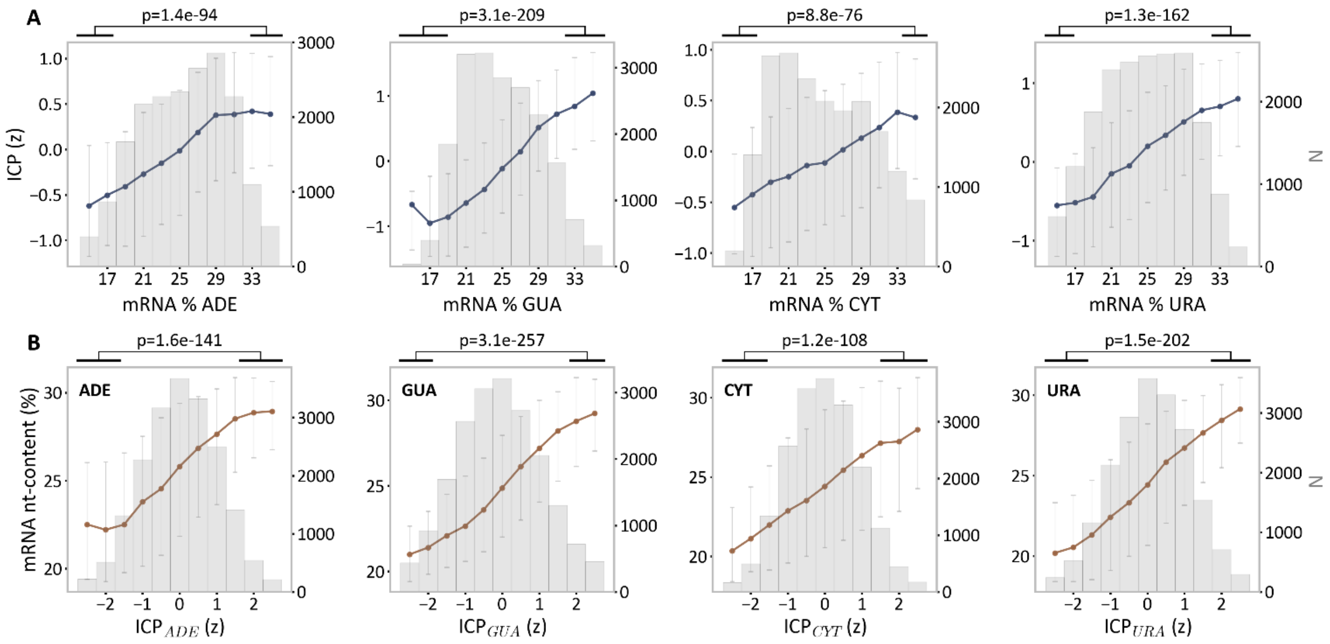


**fig. S15. *In vitro* HTR-SELEX patterns mirror the *in vivo* results.** Reproduction of Fig. S3 but using ICP values as calculated from HTR-SELEX data instead of eCLIP data. **(A)** *(left to right)* histograms of target mRNA nucleotide content (N=19,158; bin-width = 2%; right-hand y-axis) and the corresponding bin-median ICP (blue dots, left-hand y-axis), for all 4 standard RNA nucleotides. **(B)** histograms of mRNA ICP (N=19,158; bin-width = 0.5, right-hand y-axis) and the corresponding bin-median mRNA nucleotide content (orange dots, left-hand y-axis), for all 4 standard RNA nucleotides. Error bars represent the associated interquartile ranges. The reported p-values were calculated for the top and the bottom 1000 mRNAs in each panel using the 2-sided Mann-Whitney U-test, whereby the corresponding x-axis ranges are indicated by the horizontal lines above.

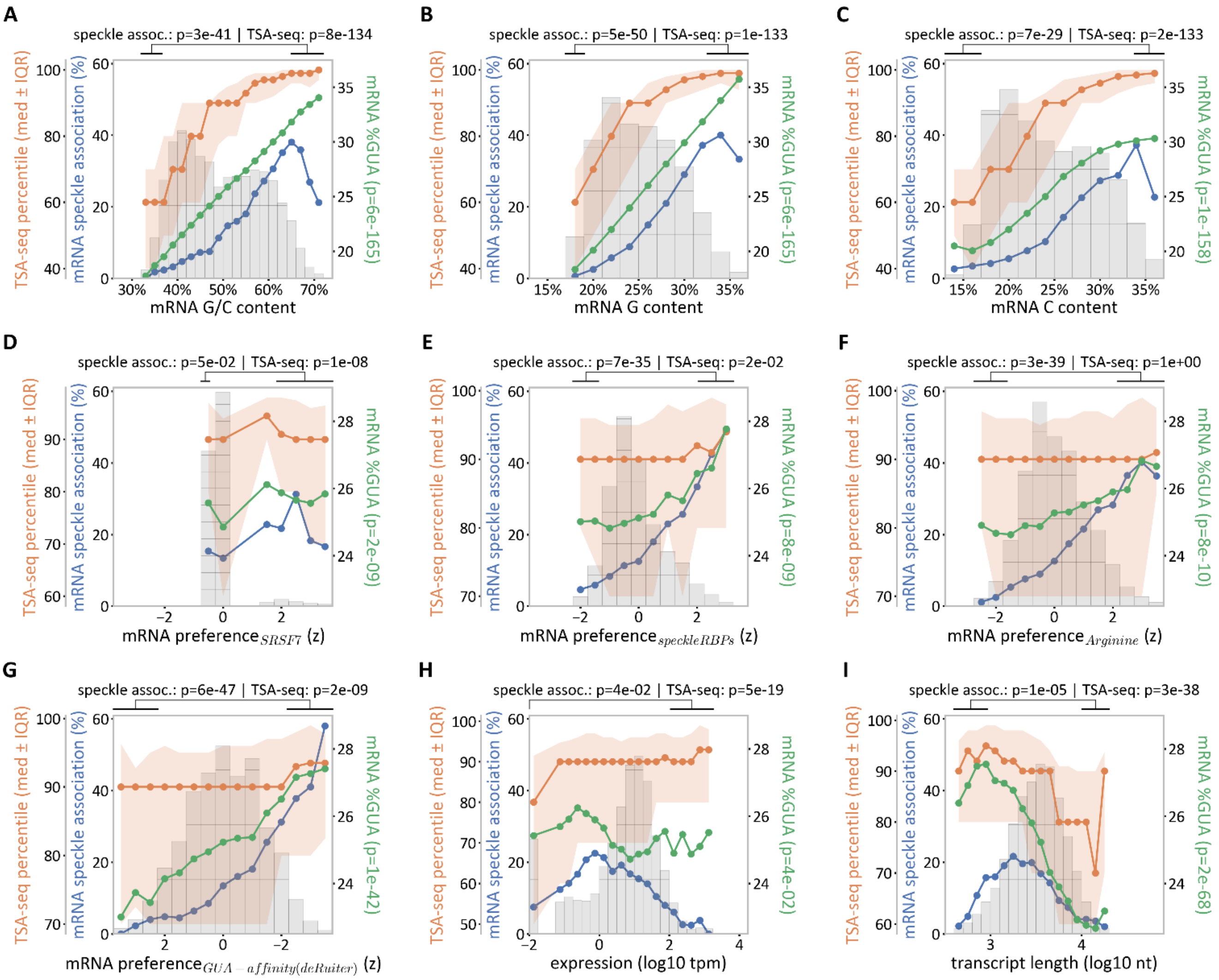


**fig. S16. Covariates of mRNA speckle association and chromatin localization (TSA-seq).** The blue line represents bin-mean mRNA speckle association (%). The orange line and corresponding shaded region represent the bin-median TSA-seq percentile and its interquartile range (IQR). The green line represents the bin-mean mRNA GUA content (%), with its p-value indicated in the right y-axis label. P-values displayed refer to the difference in speckle association and TSA-seq percentile between the bottom and top 500 mRNAs (Mann-Whitney U-test, 2-sided), with corresponding x-axis ranges indicated by horizontal lines. The grey blocks indicate the number of mRNAs in each bin (multiples of 500). The x-axes represent mRNAs binned by **(A)** MANE transcript G/C content, **(B)** G content, **(C)** C content, and **(D)** SRSF7, or binned by interaction preference for **(E)** speckle RBPs, **(F)** arginine, and **(G)** GUA-affinity (de Ruiter), alongside **(H)** expression (log10 tpm; zero-values replaced by $10^{-2}$) and **(I)** transcript length (log10 nt).
